# Speech clocks decode dementia phenotypes, social exposome, and biological aging

**DOI:** 10.1126/sciadv.aef9864

**Published:** 2026-09-30

**Authors:** Hernan Hernandez, Lizeth Katherine Pedraza, Hernando Santamaria-Garcia, Sebastian Moguilner, Agustina Legaz, Pavel Prado, Jhosmary Cuadros, Lucía Amoruso, Liset Gonzalez, Damián Dellavale, Juan Pablo Espinoza–Puelles, Javier Palma Espinosa, Cecilia Jarne, Fabio Mattiussi, Matías Caccia, Alejandro Sosa Welford, Nicolás Pelella, Jeremías Inchauspe, Franco J. Ferrante, Gonzalo Pérez, Marcelo Adrián Maito, Guido Rocatti, Maria Eugenia Godoy, Joaquin Migeot, Paulina Orellana, Ariel Caviedes, Martin Bruno, Leonel Takada, Andrea Slachevsky, Maria I Behrens, Bárbara Bruna, David Aguillon, Lina Zapata, Jose Alberto Avila-Funes, Nilton Custodio, Bruce Miller, Maria Luisa Gorno-Tempini, Stefanie Pina Escudero, Pablo Reyes, Kun Hu, Maira Okada de Oliveira, Carlos Coronel-Oliveros, Josephine Cruzat, Juan Felipe Cardona, Michael Corley, Irene B. Meier, Vaibhav A. Narayan, Enzo Tagliazucchi, Sandra Baez, Claudia Duran-Aniotz, Adolfo M. García, Agustin Ibanez

**Affiliations:** ^1^ Latin American Brain Health Institute (BrainLat), Universidad Adolfo Ibañez, Santiago de Chile, Chile.; ^2^ Laboratorio de Bases Biológicas del Comportamiento, Facultad de Ciencias del Comportamiento, Universidad de La Sabana, Chía, Colombia.; ^3^ Pontificia Universidad Javeriana (PhD Program in Neuroscience), Bogotá, San Ignacio, Colombia.; ^4^ Center of Memory and Cognition Intellectus, Hospital Universitario San Ignacio Bogotá, San Ignacio, Colombia.; ^5^ Universidad Nacional de Colombia, Bogotá, Colombia.; ^6^ Fundación Santa Fe de Bogotá, Departamento de Salud Mental, Bogotá, Colombia.; ^7^ Universidad de los Andes, Bogotá, Colombia.; ^8^ Cognitive Neuroscience Center, Departamento de Ciencias de la Vida y del Comportamiento, Universidad de San Andrés, Buenos Aires, Argentina.; ^9^ Harvard Medical School, Boston, MA, USA.; ^10^ Global Brain Health Institute (GBHI), University of California, San Francisco, CA, USA.; ^11^ Escuela de Fonoaudiología, Facultad de Ciencias de la Rehabilitación y Calidad de Vida, Universidad San Sebastián, Santiago, Chile.; ^12^ Grupo de Bioingeniería, Decanato de Investigación, Universidad Nacional Experimental del Táchira, San Cristóbal 5001, Venezuela.; ^13^ Advanced Center for Electrical and Electronic Engineering, Universidad Técnica Federico Santa María, Valparaíso 2390123, Chile.; ^14^ Basque Center on Cognition, Brain and Language (BCBL), San Sebastian, Spain.; ^15^ Ikerbasque, Basque Foundation for Science, Bilbao, Spain.; ^16^ Facultad de Ingeniería, Universidad de Concepción, Concepción, Chile.; ^17^ Consejo Nacional de Investigaciones Científicas y Técnicas (CONICET), Centro Atómico Bariloche, San Carlos de Bariloche, Río Negro, Argentina.; ^18^ Global Brain Health Institute (GBHI), Trinity College Dublin (TCD), Dublin, Ireland.; ^19^ Center for Social and Cognitive Neuroscience (CSCN), School of Psychology, Universidad Adolfo Ibáñez, Santiago, Chile.; ^20^ IRyS Group, Physical Education School, Pontificia Universidad Católica de Valparaíso, Valparaíso, Chile.; ^21^ Centro Interdisciplinario de Neurociencia de Valparaíso, Valparaíso, Chile.; ^22^ Consejo Nacional de Investigaciones Científicas y Técnicas (CONICET), Buenos Aires, Argentina.; ^23^ Universidad Nacional de Quilmes, Buenos Aires, Argentina.; ^24^ Aarhus Universitetshospital, Aarhus, Denmark.; ^25^ Universitat Pompeu Fabra, Barcelona, Spain.; ^26^ Consejo Nacional de Investigaciones Científicas y Técnicas (CONICET), Godoy Cruz, Buenos Aires, Argentina.; ^27^ Facultad de Ingeniería, Universidad de Buenos Aires, Colón, Argentina.; ^28^ Instituto de Ciencias Biomédicas (ICBM), Universidad Católica de Cuyo, San Juan, Argentina.; ^29^ Hospital das Clinicas, University of Sao Paulo Medical School, Sao Paulo, Brazil.; ^30^ Centro Gerociencias, Salud Mental y Metabolismo (GERO), Santiago, Chile.; ^31^ Laboratorio de Neuropsicología y Neurociencias Clínicas (LANNEC), Núcleo Interdisciplinario de Fisiología, Biofísica y Fisiopatología-ICBM, Departamento de Ciencias Neurológicas Oriente y Neurociencias, Facultad de Medicina, Universidad de Chile, Avenida Salvador 486, Providencia, Santiago, Chile.; ^32^ Centro de Memoria y Neuropsiquiatría (CMYN), Servicio de Neurología, Hospital del Salvador y Facultad de Medicina, Universidad de Chile, Santiago, Chile.; ^33^ Departamento de Neurología y Psiquiatría, Clínica Alemana-Universidad del Desarrollo, Santiago, Chile.; ^34^ Centro de Investigación Clínica Avanzada (CICA), Hospital Clínico Universidad de Chile, Santiago, Chile.; ^35^ Departamento de Neurología y Neurocirugía, Hospital Clínico Universidad de Chile, Santiago, Chile.; ^36^ Departamento de Neurociencia, Facultad de Medicina, Universidad de Chile, Santiago, Chile.; ^37^ Grupo de Neurociencias de Antioquia, Facultad de Medicina, Universidad de Antioquia, Medellín, Colombia.; ^38^ Fundacion Valle del Lili, Cali, Colombia.; ^39^ Universidad Icesi, Cali, Colombia.; ^40^ Dirección de Ensenanza, Instituto Nacional de Ciencias Medicas y Nutricion, Salvador Zubirán, DF, Mexico.; ^41^ Unidad de Investigación de Deterioro Cognitivo y Prevención de Demencia, Instituto Peruano de Neurociencias, Lince, Peru.; ^42^ Edward and Pearl Fein Memory and Aging Center, University of California, San Francisco (UCSF), San Francisco, USA.; ^43^ Trinity College Dublin, The University of Dublin, Dublin, Ireland.; ^44^ Edward and Pearl Fein Memory and Aging Center, Department of Neurology, Weill Institute for Neurosciences, University of California, San Francisco, CA, USA.; ^45^ Departamento de Psiquiatría y Salud Mental, Facultad de Medicina, Pontificia Universidad Javeriana, Bogotá, Colombia.; ^46^ Oficina de Investigaciones, Hospital Universitario San Ignacio, Bogotá, Colombia.; ^47^ Department of Anesthesia, Critical Care and Pain Medicine, Massachusetts General Hospital, Harvard Medical School, Massachusetts, USA.; ^48^ Division of Sleep Medicine, Harvard Medical School, Massachusetts, USA.; ^49^ Department of Neurology, Hospital das Clinicas, University of Sao Paulo School of Medicine, Sao Paulo, Brazil.; ^50^ Departamento de Biología, Facultad de Ciencias, Universidad de Chile, Santiago, Chile.; ^51^ Department of Developmental Sciences, Cognition and Neuroscience, Faculty of Psychology, Universidad del Valle, Cali, Colombia.; ^52^ Rose Stein Institute for Research on Aging and Center for Healthy Aging, UCSD, La Jolla (San Diego), USA.; ^53^ University of California San Diego Department of Medicine, Division of Geriatrics, Gerontology, and Palliative Care, UCSD, La Jolla (San Diego), USA.; ^54^ Davos Alzheimer’s Collaborative, Wayne PA, USA.; ^55^ Mental Health Goals Program, Office of Life Sciences UK Visiting Professor, Kings College, London, UK.; ^56^ University of Buenos Aires, Buenos Aires, Argentina.; ^57^ Departamento de Psicología, Universidad de los Andes, Bogotá, Colombia.; ^58^ Departamento de Lingüística y Literatura, Facultad de Humanidades, Universidad de Santiago de Chile, Santiago, Chile.; ^59^ Department of Biophysics, School of Medicine, Istanbul Medipol University, 34815 Istanbul, Türkiye.; ^60^ Barcelonaβeta Brain Research Center (BBRC), Pasqual Maragall Foundation, 08005 Barcelona, Spain.

## Abstract

Biological aging clocks offer estimations of aging and dementia, yet scalability is limited. We introduce a large-scale, cross-national speech clock derived from 2928 individuals across five Latin American countries, spanning healthy controls (HCs), mild cognitive impairment (MCI), Alzheimer’s disease (AD), and non-language/language-dominant frontotemporal dementia (nldFTD/ldFTD). Multimodal acoustic and linguistic features were trained with supervised models to estimate chronological age, generating speech age gaps (SAGs) as cross-sectional markers of deviations from chronological age, with positive values interpreted as relatively older-appearing speech profiles. SAGs differentiated diagnostic groups (HCs < patient groups, with AD < nldFTD < ldFTD). This pattern was associated with clinical/cognitive domains. SAGs correlated with phosphorylated tau (p-Tau217) in AD and social exposome in HCs and AD. Brain clocks (structural/functional/combined) were associated with SAG in AD, nldFTD, and ldFTD. Epigenetic age correlated with SAGs in HCs and AD across Hannum, Retroclock, and OMICmAge, whereas ldFTD associations were limited to Retroclock and OMICmAge. These results indicate that SAGs capture cross-sectional multilevel aging-related variation and may offer a scalable, culturally adaptable, low-cost biomarker candidate for research in underrepresented global settings.

## INTRODUCTION

Aging clocks have emerged as dimensional and transdiagnostic metrics to assess accelerated (vulnerable) or delayed (resilient) biological aging through deviations from chronological age (*1*–*7*). They offer an innovative approach to quantify individual aging trajectories, capture environmental exposures and individual lifestyles, and characterize diseases (*1*, *4*, *8*). For instance, biobehavioral age gaps (BBAGs), derived from multiple risk and protective factors, provide evidence for the impact of social exposomes on accelerated aging across the life span (*1*). Brain clocks derived from functional magnetic resonance imaging (fMRI) and electroencephalography capture discrepancies between chronological age and brain age, commonly referred to as brain age gaps (BAGs) (*5*, *7*, *9*). Epigenetic aging clocks offer similar estimations based, for example, on measures of DNA methylation (*10*). Moreover, multidimensional aging clocks (*2*, *11*) combine biobehavioral, neuroimaging, and DNA methylation data to capture diverse effects of aging-related conditions (*6*, *12*, *13*).

However, numerous gaps remain in the implementation of aging clocks in healthy elders and persons with dementia. Brain and blood-based biomarkers remain costly and logistically demanding, with limited access in low-resource settings (*2*, *14*). BBAGs rely on low-scalability instruments tapping on cognitive, functional, or other clinical dimensions, including formal tests (which require specialized clinicians for administration and scoring) and self-report surveys (which are literacy-dependent and involve bias-prone subjective ratings) (*15*). A growing need emerges for objective, automated, affordable, noninvasive aging markers enabling timely, large-scale, equitable deployment in low-income, underrepresented regions with high dementia burden (*5*, *15*–*18*).

Natural speech analysis offers a powerful, unexplored alternative (*19*). Acoustic and linguistic features are sensitive to core cognitive-motoric signatures of aging and dementia (e.g., reduced processing speed, flattened affect, and semantic memory changes). Declines in overall processing speed, a cognitive hallmark of aging and dementia, are captured by speech timing features, with longer pauses and syllables alongside lower speech rate indexing reduced word retrieval efficiency. Affect, another domain that ebbs with age and more so in dementia, is reflected in pitch and emotional content (*20*). Changes in semantic memory, including vocabulary enrichment in aging and degradation in dementia, can be captured by semantic features, such as concreteness (sensory apprehensibility of a word’s referent) (*19*, *21*) and granularity (level of semantic precision) (*22*), alongside lexical and verbosity features (including measures of overall verbal output and word-class ratios) (*23*). Automated speech and language analysis affords an objective, scalable framework to derive these features. Verbal recordings and transcriptions can be collected effortlessly, automatically, and at low cost, yielding immediate acoustic and linguistic features in multiple languages (*24*) for analysis via user-friendly apps and large language models (*25*–*27*). Many such features (e.g., speech rate, prosody, word properties, and semantic embeddings) may encode signatures of disease (*19*), proxies of cognitive reserve (*13*), and exposomic influences (*1*, *15*, *28*). Crucially, they may allow to estimate speech age gaps (SAGs; deviations between chronological and speech-predicted age), affording a scalable complement to more complex and costly data sources. To date, however, no study has examined SAGs, let alone their association with multimodal phenotypes of aging and dementia.

Inspired by well-established biological clocks (*2*, *5*, *6*, *8*), here, we introduce SAGs as an automated, scalable age-gap framework. Our sample comprises 2928 participants, enrolled through the Multi-Partner Consortium to Expand Dementia Research in Latin America (ReDLat) (*29*), spanning cognitively healthy controls (HCs) and persons with mild cognitive impairment (MCI), Alzheimer’s disease (AD), non-language-dominant frontotemporal dementia (nldFTD; comprising behavioral variant frontotemporal dementia, corticobasal syndrome, and progressive supranuclear palsy), and language-dominant frontotemporal dementia (ldFTD; comprising nonfluent agrammatic and semantic variant primary progressive aphasia). We leverage harmonized speech recordings, plasma biomarkers [with p-Tau217, the most robust marker of the AT(N) framework (*30*–*32*)], composite clinical/cognitive scores, and social exposome measures (*15*). We also examined associations between SAGs and previously developed epigenetic and brain clocks (*5*, *6*, *14*). We hypothesize that positive SAGs (accelerated aging) will index multimodal phenotypes (tau burden, clinical/cognitive outcomes, and exposome profiles) and biological aging (brain and methylation clocks) across the healthy aging and dementia continuum.

## RESULTS

The overall pipeline is shown in Fig. 1. We included 2928 Spanish-speaking participants to (A) assemble a multicountry dataset and (B to D) train age-predicting models with multimodal speech features to derive SAGs. The cohort comprised 1504 HCs and 1424 patients (24 with MCI, 1068 with AD, 255 with nldFTD, and 77 with ldFTD, recruited in Argentina (*n* = 195), Chile (*n* = 299), Mexico (*n* = 509), Peru (*n* = 798), and Colombia (*n* = 1127) (Fig. 1A, Table 1, and table S1). We implemented a validated feature-extraction pipeline (Fig. 1B) (*26*, *27*) to derive interpretable acoustic and linguistic predictors, categorized into speech timing, pitch-related, semantic (concreteness and granularity), lexical, emotional, and verbosity features. We then fit supervised regression models to predict chronological age from these predictors, using *k*-fold cross-validation with hyperparameter tuning (Fig. 1C). From the predicted ages, we computed SAGs as predicted age − chronological age; SAG > 0 indicates a relatively older-appearing speech profile, and SAG < 0 indicates a relatively younger-appearing or preserved speech profile (Fig. 1D). Model performance and predictor importance were derived from speech features (Fig. 1E.1) and group statistical analyses of SAGs (HCs, MCI, AD, nldFTD, and ldFTD) (Fig. 1E.2). We tested associations between SAGs and previously well-established biological clocks and phenotypic biomarkers (Fig. 1F): plasma p-Tau217 (F.1); clinical/cognitive assessments targeting functional abilities, attention and executive functioning, global cognition, and multiple memory domains—including episodic, visual, verbal, and nonverbal memory (F.2); social exposome (F.3); BAGs derived from structural, functional, and combined neuroimaging (F.4); and epigenetic (Hannum, OMICmAge, and Retroclock) clocks (F.5).

**Fig. 1.**
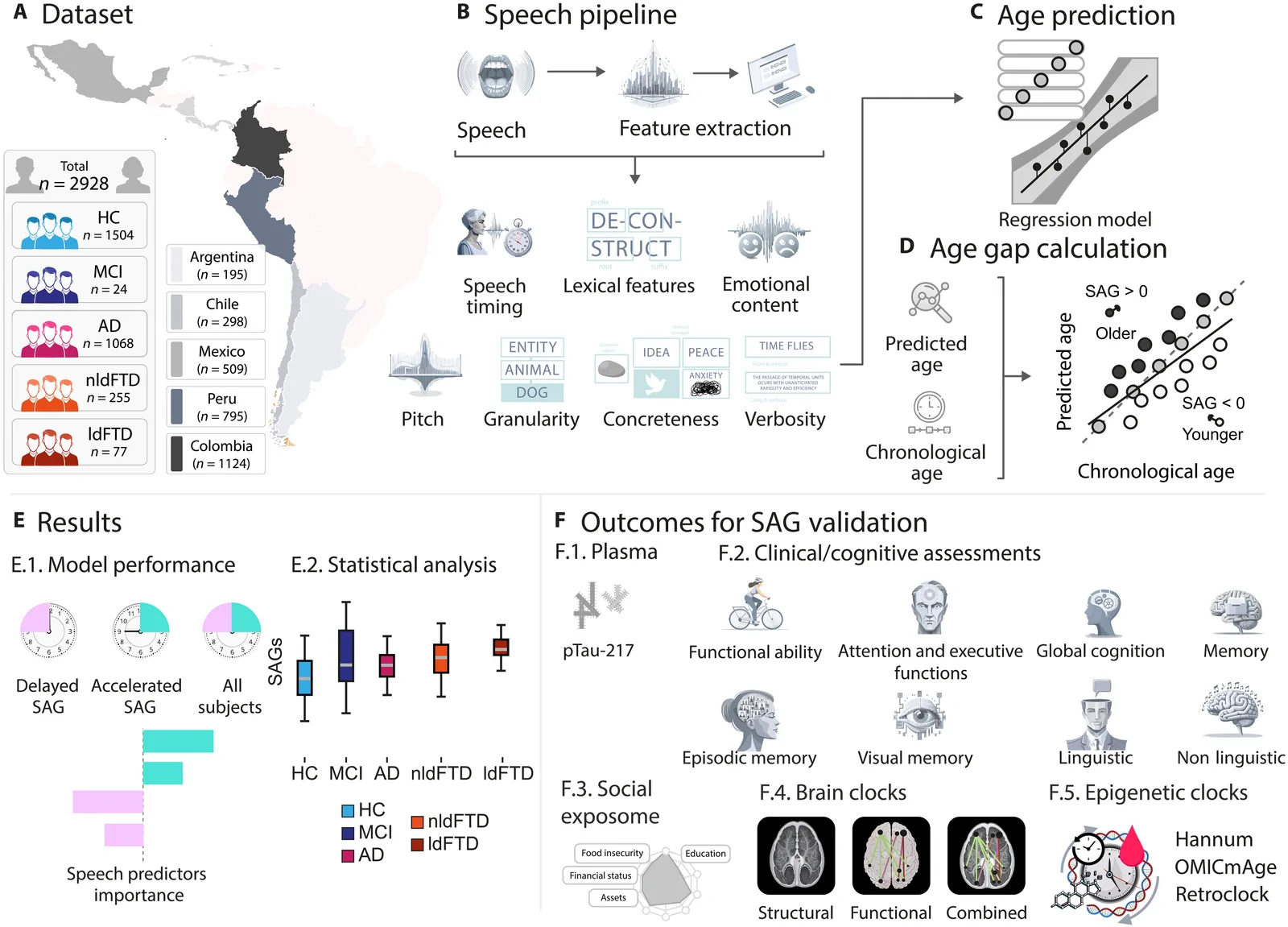
Study design. (**A**) We recruited 2928 participants from five Latin American countries (Argentina, Chile, Mexico, Peru, and Colombia), encompassing HCs and persons with MCI, AD, and both non-language-dominant and language-dominant variants of frontotemporal dementia degeneration (nldFTD and ldFTD). (**B**) Speech recordings were processed through a standardized feature-extraction pipeline to derive interpretable acoustic and linguistic predictors, categorized into speech timing, pitch-related, semantic (concreteness and granularity), lexical, emotional, and verbosity features. (**C**) Supervised regression models were trained to predict chronological age from these multimodal speech features using *k*-fold cross-validation. (**D**) SAGs were computed for each participant as predicted age minus chronological age, such that SAG > 0 reflects accelerated linguistic aging and SAG < 0 reflects delayed or preserved aging. (**E**) We quantified model performance and feature importance across speech-predictor categories (E.1) and performed group-level statistical analyses of SAGs across HC, MCI, AD, nldFTD, and ldFTD (E.2). (**F**) Associations between SAGs and independent, well-established clocks and biomarkers were examined, including plasma p-Tau217 levels (F.1); clinical and cognitive assessments covering linguistic, nonlinguistic, functional ability, attention and executive functioning, global cognition, and memory domains (episodic and visual) (F.2); MSE (F.3); brain clocks derived from structural, functional, and combined neuroimaging (F.4); and epigenetic (Hannum, OMICmAge, and Retroclock) clocks (F.5).

**Table 1.** Demographic characterization of subjects. Demographic information of the study cohort, including participant distribution by country, age, and sex across diagnostic groups.

Sample size		HCs	MCI	AD	nldFTD	ldFTD	Statistics
		1504	24	1068	255	77	
Country	Argentina	77		104	11	3	
	Chile	158	2	101	29	9	
	Colombia	403	22	558	100	44	
	Mexico	353		129	23	4	
	Peru	513		176	92	17	
Age, years [mean (SD)]		60.90 (11.18)	65.67 (14.16)	70.98 (7.96)	66.34 (8.01)	66.45 (7.39)	*F* (4, 2923) = 166.79, *P* < 0.001, η^2^ = 0.186
Sex (female:male)		1095:409	16:8	697:371	126:129	40:37	χ^2^ (4, *N* = 2928) = 68.17, *P* < 0.001

### SAG building using speech composite features

We first investigated whether speech-derived composites can predict age-related variance. Chronological age was successfully predicted from the seven composite categories [coefficient of determination (*R*^2^) = 0.44, *f*^2^ = 0.80, *r* = 0.67, root mean squared error (RMSE) = 11.05, mean absolute error (MAE) = 9.04, and mean directional error (MDE) = −0.08; Fig. 2A]. Feature importance mean decrease in impurity (MDI) indicated that verbosity and speech timing were the most important categories, consistent with delayed aging (lower predicted age), whereas pitch and emotional content were the dominant contributors to accelerated aging (higher predicted age). Further analyses showed moderate alignment for uncorrected predictions (*r* = 0.44) and tighter alignment after bias correction (*r* = 0.74; Fig. 2A). These results suggest that composite speech features capture an interpretable aging signal suitable for the prediction of SAGs.

**Fig. 2.**
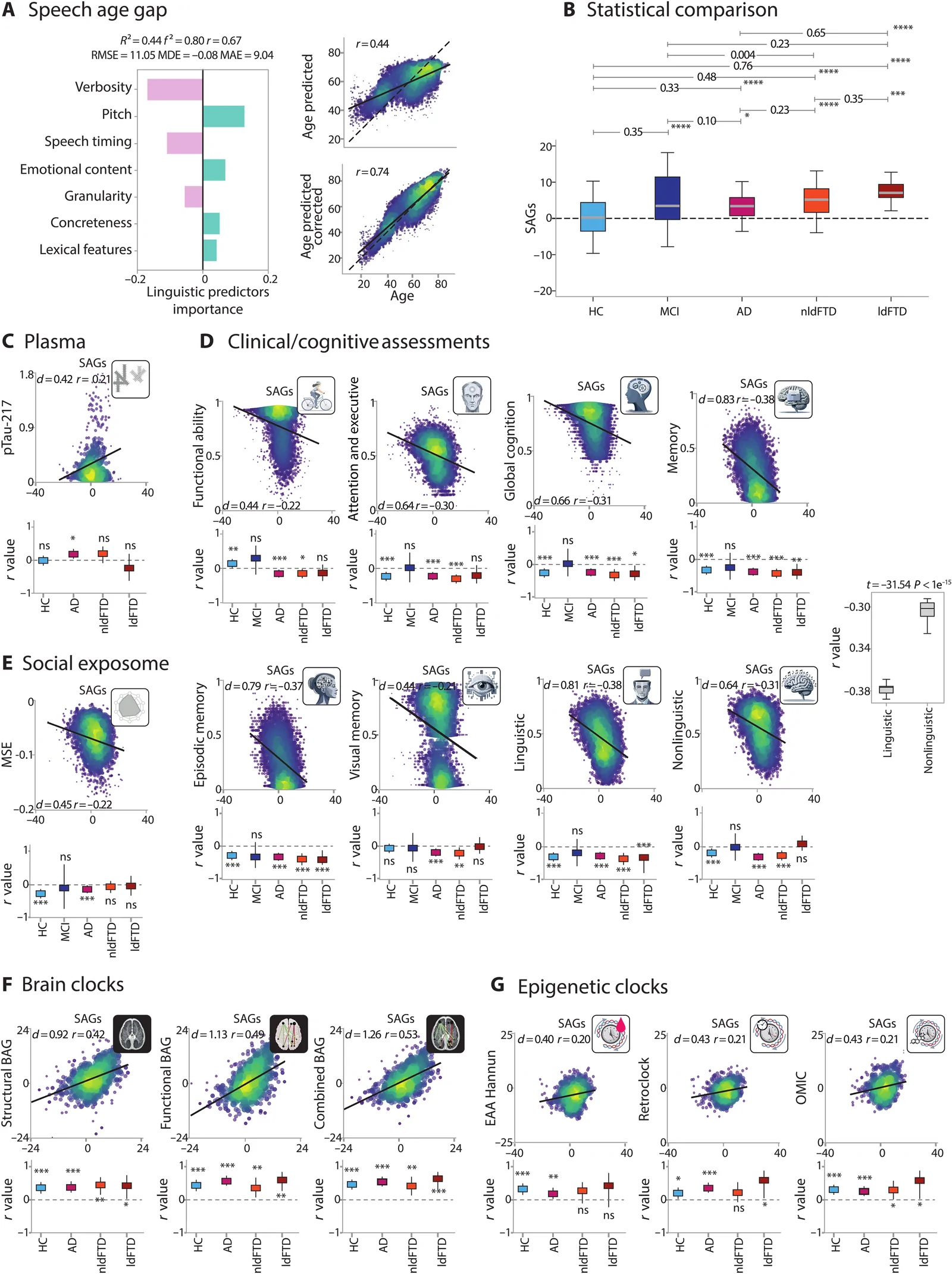
Main results. (**A**) SAG performance and feature importance across speech domains. Verbosity, timing, and granularity indexed delayed/preserved aging, whereas pitch, emotion, and concreteness mainly indexed accelerated aging (*R*^2^ = 0.44, *f*^2^ = 0.80, *r* = 0.67, RMSE = 11.05, MAE = 9.04, and MDE = −0.08). Scatterplots show uncorrected/bias-corrected predicted versus chronological age. (**B**) SAGs differed across HC, MCI, AD, nldFTD, and ldFTD; neurodegenerative groups showed accelerated aging versus HC (δd = 0.33 to 0.76, all *P* < 1 × 10^−5^). (**C**) Greater SAGs related to higher plasma p-Tau217 (*r* = 0.21; *d* = 0.42; *P* = 3.30 × 10^−6^), coupling in AD. (**D**) Larger SAGs tracked poorer cognition, memory, episodic, and visual memory (*r* = −0.21 to −0.38; *d* = 0.44 to 0.81). AD and nldFTD showed negative SAG-cognition associations, whereas HC, MCI, and ldFTD showed domain-specific patterns. Composite analyses showed negative correlations across linguistic/nonlinguistic domains, strongest for linguistic tasks (*t* = −31.54, *P* < 1 × 10^−15^). (**E**) SAGs were associated with the MSE, with greater SAGs linked to a more adverse profile (*r* = −0.22; *d* = 0.45; *P* = 2.10 × 10^−21^), significant in HC and AD. (**F**) SAGs correlated with structural, functional, and combined BAGs (*r* = 0.42; *d* = 0.92; *P* = 1.95 × 10^−28^; *r* = 0.49; *d* = 1.13; *P* = 2.56 × 10^−39^; *r* = 0.53; *d* = 1.26; *P* = 4.25 × 10^−47^), with positive coupling in HC, AD, nldFTD, and ldFTD. (**G**) SAGs aligned with epigenetic acceleration: Hannum (*r* = 0.20; *d* = 0.40; *P* = 3.29 × 10^−5^), Retroclock (*r* = 0.21; *d* = 0.43; *P* = 9.66 × 10^−6^), and OMICmAge (*r* = 0.21; *d* = 0.43; *P* = 2.14 × 10^−5^), with HC and AD effects. Points represent participants; black lines indicate regression fits; shaded areas indicate 95% confidence intervals (CIs). Extended group- and domain-specific results are provided in text S1. ns, not significant.

### SAGs capture diagnosis categories

We analyzed whether SAGs differentiate between HCs and patient groups, using a permutation-based test with 10,000 iterations. Because positive SAG index accelerated aging, we expected patients to exhibit larger SAGs than HCs. This pattern was confirmed: HCs had lower SAGs than every patient group [Cliff’s delta (δd) = 0.33 to 0.76, all *P* < 1 × 10^−5^; Fig. 2B], evidencing a more pronounced accelerated aging in patients. Within patient cohorts, ldFTD showed the greatest SAGs, surpassing AD (δd = 0.65, *P* < 1 × 10^−5^) and nldFTD (δd = 0.35, *P* = 2 × 10^−4^). SAGs were greater for nldFTD than for AD (δd = 0.23, *P* < 1 × 10^−5^) (Fig. 2B and table S2). We also examined whether SAGs differentiate between HCs and patient groups without data augmentation and using a leave-one-country-out (LOCO) approach. In both cases, significant differences were observed in the same groups, with comparable effect sizes to the main results (fig. S1A).

### Plasma biomarker p-Tau217

We examined the predictive value of SAGs using previously established phenotypic markers associated with neurodegeneration. SAGs were positively associated with plasma p-Tau217 (*r* = 0.21; *d* = 0.42; *P* = 3.30 × 10^−6^; Fig. 2C). Within groups, analyses showed a positive association in patients with AD (*r* = 0.18, *P* = 0.01). No other individual associations were observed in the HC, nldFTD, or ldFTD groups (Fig. 2C and table S3).

### SAGs and clinical/cognitive domains

Across clinical/cognitive domains, accelerated aging tracked worse cognition (*r* = −0.21 to −0.38; *d* = 0.44 to 0.81) (Fig. 2D). At the diagnostic-group level, only AD and nldFTD exhibited consistent negative associations between SAGs and performance across all clinical and cognitive domains assessed. In AD, accelerated aging was associated with poorer performance in all domains: functional ability (*r* = −0.12, *P* = 2.74 × 10^−5^), executive function (*r* = −0.23, *P* = 4.94 × 10^−15^), global cognition (*r* = −0.23, *P* = 1.06 × 10^−14^), overall memory (*r* = −0.34, *P* = 5.59 × 10^−31^), episodic memory (*r* = −0.33, *P* = 2.08 × 10^−28^), and visual memory (*r* = −0.17, *P* = 1.67 × 10^−8^). In nldFTD, we observed a similar pattern with significant negative associations for functional ability (*r* = −0.14, *P* = 0.025957), executive function (*r* = −0.30, *P* = 5.55 × 10^−7^), global cognition (*r* = −0.31, *P* = 3.47 × 10^−7^), overall memory (*r* = −0.42, *P* = 3.83 × 10^−12^), episodic memory (*r* = −0.39, *P* = 6.40 × 10^−11^), and visual memory (*r* = −0.20, *P* = 0.001236). The strongest effects in both groups were observed for memory outcomes. Together, these patterns indicate that SAGs reflect broad cognitive aging beyond domain-specific variance. Extended data for each group-by-domain combination are provided in table S4.

### SAGs and linguistic and nonlinguistic domains

Complementary analyses compared SAGs against aggregates derived from the clinical/cognitive assessment, categorized into linguistic (aggregating all tasks with explicit verbal components) and nonlinguistic (aggregating tasks independent of verbal processing) composites, revealing differential associations. Larger SAGs were associated with poorer linguistic (*r* = −0.38; *d* = 0.81; *P* = 2.63 × 10^−98^) and nonlinguistic (*r* = −0.31; *d* = 0.64; *P* = 9.14 × 10^−64^) outcomes (Fig. 2D and table S5). As expected, the coupling was stronger for linguistic than for nonlinguistic tasks (*t* = −31.54, *P* < 1 × 10^−15^). Within-group analyses showed negative associations between SAGs and linguistic performance in HCs (*r* = −0.31, *P* = 1.48 × 10^−35^), AD (*r* = −0.27, *P* = 1.30 × 10^−19^), nldFTD (*r* = −0.40, *P* = 2.84 × 10^−11^), and ldFTD (*r* = −0.35, *P* = 0.0015), whereas no significant associations were detected for the MCI group. Nonlinguistic composites showed the same direction in HCs (*r* = −0.17, *P* = 4.41 × 10^−12^), AD (*r* = −0.28, *P* = 1.59 × 10^−21^), and nldFTD (*r* = −0.30, *P* = 4.79 × 10^−7^), with no associations observed for the MCI and ldFTD groups. These results indicate that SAGs provide a generalizable indicator of performance across linguistic and nonlinguistic domains. The associations between SAGs and individual clinical/cognitive assessments followed a similar pattern to the main results, although effect sizes were slightly smaller (table S6).

### Social exposome

A multimodal social exposome (MSE) (*15*) captures a multidimensional factor that integrates life span social determinants—such as education, food insecurity, financial status, access to healthcare, and early-life experiences—which collectively shape dementia risk and brain health (*1*, *15*). Larger SAGs were associated with adverse social exposome (*r* = −0.22; *d* = 0.45; *P* = 2.10 × 10^−21^; Fig. 2E and table S7). In within-group analyses, this association was significant in HCs (*r* = −0.28, *P* = 1.83 × 10^−19^) and patients with AD (*r* = −0.13, *P* = 0.00027). No other individual associations were observed for the MCI, nldFTD, or ldFTD groups.

### SAGs and biological aging

For the brain and epigenetic clock analyses (Fig. 2, F and G), we only included diagnostic groups for which participants had complete speech production tasks and the corresponding clock-specific measures at the individual level. Because no MCI participants had both complete speech data and corresponding brain or epigenetic clock measures, this group could not be included in the within-group analyses.

#### Brain clocks

We assessed correlations between SAGs and BAGs derived from structural (T1-MRI), functional (rs-fMRI), and combined neuroimaging models. Across the cohort, SAGs correlated positively with structural (*r* = 0.42; *d* = 0.92; *P* = 1.95 × 10^−28^), functional (*r* = 0.49; *d* = 1.13; *P* = 2.56 × 10^−39^), and combined (*r* = 0.53; *d* = 1.26; *P* = 4.25 × 10^−47^) BAGs (Fig. 2F and table S8), indicating that greater speech-predicted accelerated aging aligns with brain clocks. Within-group analyses further supported the positive association between SAGs and BAGs across diagnostic categories. Accelerated aging was associated with larger functional BAGs across all groups: HCs (*r* = 0.44, *P* = 5.06 × 10^−9^), AD (*r* = 0.57, *P* = 4.18 × 10^−26^), nldFTD (*r* = 0.37, *P* = 0.0068), and ldFTD (*r* = 0.60, *P* = 0.0010). A similar pattern was observed for structural BAGs in HCs (*r* = 0.38, *P* = 5.87 × 10^−7^), AD (*r* = 0.40, *P* = 1.61 × 10^−12^), nldFTD (*r* = 0.42, *P* = 0.0016), and ldFTD (*r* = 0.43, *P* = 0.02599). For the combined BAGs, positive correlations with SAGs were again present in HCs (*r* = 0.47, *P* = 3.44 × 10^−10^), AD (*r* = 0.58, *P* = 8.3 × 10^−28^), nldFTD (*r* = 0.43, *P* = 0.0014), and ldFTD (*r* = 0.62, *P* = 0.0006). Thus, across diagnoses, participants with larger SAGs showed accelerated BAGs, supporting the biological embedding of speech-based aging metrics.

#### Epigenetic clocks

SAGs were associated with epigenetic age acceleration (EAA) using three clocks: Hannum (*r* = 0.20; *d* = 0.40; *P* = 3.29 × 10^−5^), Retroclock (*r* = 0.21; *d* = 0.43; *P* = 9.66 × 10^−6^), and OMICmAge (*r* = 0.21; *d* = 0.43; *P* = 2.14 × 10^−5^) (Fig. 2G and table S9). For group-level analyses of the association between SAGs and epigenetic aging clocks (Hannum, Retroclock, and OMICmAge), only HCs and patients with AD showed consistent positive correlations across all three measures. In HCs, accelerated aging was associated with epigenetic aging estimates on the Hannum clock (*r* = 0.31, *P* = 9.86 × 10^−5^), Retroclock (*r* = 0.20, *P* = 0.0142), and OMICmAge (*r* = 0.32, *P* = 6.56 × 10^−5^). In AD, SAGs also correlated positively with Hannum (*r* = 0.19, *P* = 0.0058), Retroclock (*r* = 0.36, *P* = 1.49 × 10^−7^), and OMICmAge (*r* = 0.29, *P* = 3.33 × 10^−5^). For the ldFTD group, a positive association was found with Retroclock (*r* = 0.57, *P* = 0.0484) and OMICmAge (*r* = 0.60, *P* = 0.0378), indicating that individuals with accelerated aging tend to show more advanced epigenetic aging.

### SAGs outperform individual speech-feature domains in diagnostic discrimination

We tested whether speech predictors could differentiate diagnostic groups after adjusting for chronological age, education, and sex. SAGs outperformed isolated feature domains across all clinical contrasts (HC versus AD, HC versus nldFTD, and HC versus ldFTD; Fig. 3A). Among the domains, linguistic and semantic-memory features yielded stronger differentiation than acoustic features. This distinction was particularly notable in ldFTD analyses, mainly driven by linguistic features—arguably reflecting this group’s language-dominant phenotype. While aggregating top-ranked linguistic and acoustic features improved performance relative to single features, it still did not match the discriminative power of the full SAG composite (Fig. 3A). These results indicate that the composite SAG framework enhances group differentiation beyond the diagnostic information carried by individual or simply aggregated speech predictors.

**Fig. 3.**
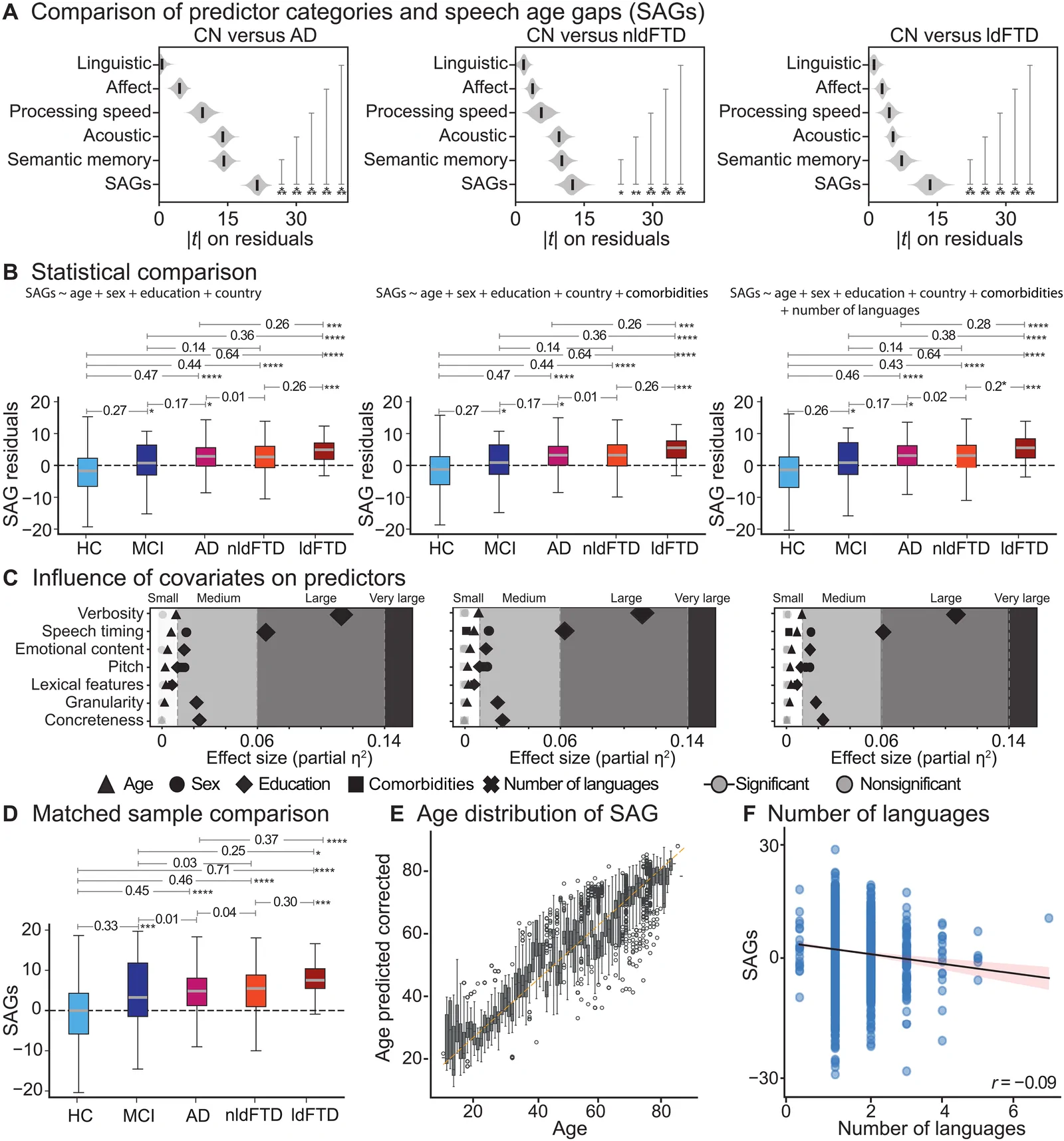
Sensitivity analyses. (**A**) Comparing predictor categories and SAGs across diagnostic contrasts. Violin plots depict the bootstrap distributions (*n* = 1000) of absolute *t* statistics (|*t*|) derived from group comparisons on demographically adjusted residuals (age, sex, and education) for each predictor category. Three diagnostic contrasts show HCs versus patients with AD, HCs versus patients with nldFTD, and HCs versus patients with ldFTD. Predictor categories include linguistic, affective, processing speed, acoustic, and semantic memory domains, alongside SAGs. (**B**) Statistical comparison of SAGs across diagnostic groups under different adjustment models. Boxplots show the distribution of demographically adjusted SAG residuals across diagnostic groups (HCs, MCI, AD, nldFTD, and ldFTD). Three models are presented: adjusted for age, sex, education, and country (left); additionally adjusted for comorbidities (middle); and further adjusted for number of languages spoken (right). (**C**) Influence of covariates on linguistic predictors. Partial η^2^ effect sizes from ANCOVA models quantifying the contribution of demographic and clinical covariates (age, sex, education, country, comorbidities, and number of languages) to each linguistic predictor. (**D**) Matched sample comparison of SAGs. Boxplots show the distribution of SAG residuals across diagnostic groups in a matched sample controlling for key demographic variables (age, sex, and education). (**E**) Age distribution of predicted SAGs. Relationship between chronological age and predicted (bias-corrected) speech age across the full sample. The dashed diagonal line indicates perfect agreement between predicted and chronological age. (**F**) Association between number of languages spoken and SAGs. Scatterplot showing the association between the number of languages spoken and SAG values, with the fitted linear trend (solid line) and 95% CI (shaded area).

### Sensitivity models confirm diagnostic differences in SAGs

To further address the potential influence of sex, comorbidities, and other demographic factors on SAG estimates, we implemented three progressive analysis of variance (ANOVA) sensitivity models. Model 1 (Primary Model) included clinical diagnosis, sex, country, education, and age as covariates. Model 2 (Covariate-Adjusted Model) extended Model 1 by incorporating a composite comorbidity score, selected because individual comorbidity variables lacked sufficient statistical power. Model 3 (Complete Sensitivity Model) further added the number of languages spoken as an additional control variable (Fig. 3B and table S10). Across all three models, the effect of clinical diagnosis remained highly significant (*P* < 1 × 10^−15^), confirming the robustness of SAG differences across diagnostic groups. The composite comorbidity score (Model 2, *F* = 3.18, *P* = 0.075) and the number of languages spoken (Model 3, *F* = 0.30, *P* = 0.58) did not substantially alter the main clinical effects, further supporting the independence of the SAG signal from these potential confounders.

We further assessed the influence of individual covariates on the linguistic predictors that contribute to the SAG composite. As depicted in the covariate influence plots, education exhibited the largest effect size (partial η^2^) on speech predictors consistent with the expected influence of socio-educational habits on discourse structure. Sex showed a small but significant effect primarily on pitch-related features. Age effects were consistently small across linguistic predictors. The composite comorbidity score and number of languages spoken showed negligible effects on most linguistic features (Fig. 3C).

We performed propensity score matching on age, sex, and education across all pairwise diagnostic comparisons (e.g., HCs versus patients with AD, HCs versus patients with nldFTD, and HCs versus patients with ldFTD). Although this procedure necessarily reduced the sample size to achieve demographic balance, the core SAG effects were fully preserved (Fig. 3D). To assess the sensitivity of these results to different matching stringency levels, we repeated the analysis using an interquartile range threshold of 1.5. As shown in the matched-sample boxplots, the diagnostic ordering of SAG values remained consistent, with effect sizes (Cliff’s delta) remaining substantial for all clinical contrasts, particularly for HCs versus patients with ldFTD.

### Bias correction, multilingualism, and network-level brain-age convergence

Model performance was further examined using the predicted-versus-observed age plot, which showed that predictions closely tracked the identity line across the full age range, with no evidence of systematic overestimation in younger participants or underestimation in older participants (Fig. 3E). The residual distribution was approximately uniform across age, providing evidence against regression-to-the-mean artifacts and confirming that the bias-correction procedure effectively mitigated age-dependent model bias.

Analyses conducted in the subset of participants with available language-background data examined the association between the number of languages spoken and SAGs. A weak negative association was observed between multilingualism and SAG values (*r* = −0.09, slope = −1.21), indicating that participants who reported speaking a greater number of languages tended to show lower speech-age estimates (Fig. 3F). Individuals reporting three or four languages showed a tendency toward lower SAG values compared with monolingual participants. Although this association did not reach conventional statistical significance in the present cross-sectional sample, its direction is consistent with the hypothesis that multilingualism may confer a protective effect against accelerated speech aging and aligns with recent evidence linking multilingualism to reduced biological aging (*13*).

To assess whether SAGs were specifically associated with accelerated aging at the whole-brain level, or instead reflected more circumscribed functional systems and brain subdomains, we tested its association with BAGs derived from canonical resting-state networks, including the default mode, dorsal attention, salience, visual, motor, and language networks (*1*, *33*). As shown in fig. S2A, the network-level BAG analysis revealed significant associations with a global language network (*34*) and a verbal production network (*35*), although to a lesser extent than whole-brain effects. Regarding other control networks, the salience network showed a smaller but significant association with SAGs (*d* = 0.22; *r* = 0.11; *P* = 0.030), consistent with its role in speech monitoring, attentional allocation during language production, and the integration of interoceptive signals relevant to affective prosody. The remaining networks (DMN, EN, MN, and VN) did not reach statistical significance, supporting the view that the speech clock is preferentially coupled to brain systems involved in accelerated and delayed aging, and secondarily to language and salience processed.

## DISCUSSION

This study introduces a large-scale speech clock and shows that speech features capture accelerated or preserved aging across healthy individuals and major dementia syndromes. Using a harmonized sample of 2928 participants from five Latin American countries, we trained a multimodal speech-based age-prediction model and computed SAGs as the core aging metric. SAGs distinguished among diagnostic groups: HCs showed the smallest gaps, followed by progressively larger values in persons with MCI, AD, and nldFTD, while those with ldFTD exhibited the greatest accelerated aging. Beyond diagnostic stratification, SAGs showed systematic convergence with independent biological and phenotypic markers. Larger SAGs were associated with higher plasma p-Tau217 when considering all participants, and within-group analyses showed a positive association in patients with AD, suggesting a disease-related coupling between tau pathology and speech-based aging. Larger SAGs were associated with poorer performance across clinical/cognitive domains (including differential patterns for linguistic and nonlinguistic outcomes) as well as with more adverse MSE profiles, notably in HCs and persons with AD. SAGs tracked biological aging across multiple clocks: They correlated with structural, functional, and combined brain-age gaps in HCs and all dementia groups, with the strongest effects in AD and ldFTD, and showed consistent positive associations with multiple DNA-methylation clocks (Hannum, Retroclock, and OMICmAge), especially in HCs and AD. Together, these findings indicate that speech clocks encode multilevel aging signatures that are coherent across clinical syndromes, biomarker modalities, cognitive dimensions, and social determinants, positioning speech as a scalable, noninvasive, and culturally adaptable marker for large-scale aging surveillance, early detection, and precision-prevention in underrepresented settings.

Extending prior work on biological clocks (*1*, *5*, *6*, *15*), our findings suggest that speech is not merely a surface-level cognitive marker, but rather a functional expression of systemic aging (*9*), integrating biological, motor, cognitive, semantic, affective, and social processes across the life span (*28*). Unlike traditional clocks that rely on neuroimaging or blood-based molecular profiles, SAGs capture ecologically grounded information, opening a robust avenue for aging-clock research at large. By targeting interpretable acoustic and linguistic features, our framework provides clinically meaningful insights. Unlike stock metrics developed for other purposes or black-box embeddings, speech timing, affective (pitch and emotion) (*36*), and lexico-semantic features (*20*, *22*) reflect core dynamics of aging and dementia, respectively, including reduced processing speed, flattened affect, and semantic memory changes. These patterns can be directly linked to core domains in standard assessments, potentially informing clinical decision-making beyond traditional tools. Moreover, they offer important translational advantages over biobehavioral, brain-based, and omics-based clocks, as they are low-cost, automatized, and susceptible of remote acquisition without specialized personnel (*19*, *26*, *27*). The convergence between SAGs, tau pathology, cognitive performance, social exposome, MRI, and methylation-based clocks supports the existence of a latent aging factor manifesting in synchrony across molecular, neural, behavioral, and linguistic levels. SAGs’ stronger convergence with brain-based than with epigenetic clocks may indicate partial organ specificity. Because speech emerges from the coordinated operation of linguistic, executive, auditory, and motor systems, SAGs may more directly reflect brain-level aging processes than whole-body methylation signals. At the same time, the significant associations with multiple epigenetic clocks suggest that SAGs are not exclusively brain-specific but may also index broader systemic aging influences expressed through neural and behavioral output. Network-specific clocks showed no significant associations with SAGs, except in the language and salience networks (but these were smaller than whole-brain effects associated with BAGs). Thus, SAGs primarily capture accelerated aging within whole-brain aging-related systems, with smaller contributions from language and salience processes. These findings are consistent with previous network-specific clocks, showing that markers of accelerated or delayed brain aging are more strongly linked to global aging-related brain patterns than to individual resting-state networks (*37*). Previous speech-based studies have typically focused on single diseases, single centers, or narrow acoustic or lexical features (*19*). The comparison with speech predictors showed that SAGs provided the strongest discrimination across all clinical contrasts, outperforming individual linguistic, semantic-memory, and acoustic domains. This finding supports the added value of the composite SAG framework, while the stronger linguistic differentiation in ldFTD is consistent with this group’s language-dominant profile. Here, we show that a speech-derived aging marker is robust across a diverse multicentric cohort, distinct dementia syndromes, and independent biological domains. This indicates that linguistic aging serves as a coherent and interpretable readout of multisystem aging that transcends specific diagnostic boundaries and site-related variability, rather than being driven by disease-specific features alone. Furthermore, the sensitivity of SAGs to social adversity underscores the fact that aging is shaped not only by biological processes but also by cumulative environmental and socioeconomic exposures, and that speech may be an efficient behavioral marker to capture these long-term influences.

Several strengths support the robustness and translational potential of this framework. This is a large study integrating speech with plasma biomarkers, detailed neuropsychology, an MSE, structural and functional brain clocks, and epigenetic clocks within a single analytic architecture. The large sample size and geographic diversity—nearly 3000 participants spanning five Latin American countries—offers robust insights on populations that have been historically underrepresented in dementia research (*17*, *29*). The speech-processing and modeling pipeline is standardized, automated, and multimodal, ensuring harmonized data processing across diverse acquisition centers through a highly scalable framework. The inclusion of advanced MRI-based and DNA-methylation aging clocks provides evidence for biological embedding of SAG beyond speech and isolated clinical correlations (*5*, *9*, *38*). The associations of MSE and SAGs link aging clocks to environmental and socioeconomic determinants. Last, the low cost and noninvasive nature of speech recordings make this approach particularly suitable for low-resource contexts and global initiatives, in which traditional biomarkers are difficult to obtain at scale (*39*).

Several limitations must be acknowledged. The design is primarily cross-sectional, which precludes causal inference and limits our ability to quantify within-person aging trajectories or predict future clinical conversion. Although positive SAGs were associated with multiple adverse clinic-biological features linked to accelerated aging, the present design does not establish whether these deviations reflect dynamic acceleration of aging over time. Longitudinal studies will be required to determine the prognostic stability, temporal progression, and clinical predictive utility of SAGs. Although we implemented harmonized procedures, multicentric variability in recording conditions, microphones, and speech prompts may introduce residual noise in the linguistic features. Automated speech-feature extraction tools may embed dialectal or cultural biases, warranting systematic evaluation (*40*). Sex, medical comorbidities, and mood-related fluctuations are likely to influence speech production and may therefore contribute to SAG variability. Our sex-adjusted sensitivity analyses indicated that the main group-level patterns persist after accounting for sex. Future work should model comorbidities and mood explicitly to distinguish nuisance variance from clinically meaningful signal. We caution against automatically regressing these factors out in all cases, as they may constitute part of the biological or behavioral phenotype (accelerated aging) expressed through speech. Sample size was limited for some diagnostic groups (especially MCI and ldFTD) and in specific biomarker subsets, reducing statistical power for subgroup analyses and potentially obscuring subtle effects. Biomarker and exposome data were not available for all participants, raising the possibility of selection biases in the multimodal convergence analyses. Last, our data are restricted to Latin American cohorts and relatively structured speech tasks. While our results underscore the robustness of SAGs, generalization onto other languages needs to be empirically established. Linguistic phenotypes of AD, for example, differ depending on the patients’ language, with specific acoustic features (namely, speech timing) generalizing between languages better than lexico-semantic features both for disease detection and severity prediction (*19*, *21*). Cross-linguistic designs should be performed in future SAG research, ideally using language-specific normalization or multilingually trained models to favor comparability (*41*). An additional factor that warrants systematic consideration in future work is multilingualism (*13*). Language experience may shape cognitive reserve, lexical access, and speech organization and could therefore modulate SAG estimates. Recent evidence indicates that multilingualism is associated with protection against accelerated aging across large cross-sectional and longitudinal cohorts (*13*). Although analyses on the subset of participants with available language background data revealed a negative association between the number of languages spoken and the SAGs, these effects showed a trend only. Because harmonized bilingualism data were not uniformly available across the multicentric cohort, this variable represents an important target for future studies. Validation in other languages, cultures, and more naturalistic conversational contexts will be important to confirm the generalizability and robustness of SAGs.

In conclusion, SAGs provide a biologically meaningful, scalable, and culturally adaptable measure of aging that aligns with cognitive performance, fluid biomarkers, social exposome, neuroimaging-derived brain clocks, and epigenetic aging. Findings suggest that speech clocks afford a shortcut to multimodal aging in health and disease that can be leveraged for early detection, equitable surveillance, and precision-prevention strategies in diverse populations. This framework opens new avenues for integrating speech clocks into global aging research, clinical workflows, and public-health initiatives aimed at improving dementia prevention and promoting brain-health equity across sociocultural contexts.

## MATERIALS AND METHODS

### Participants

The total dataset comprised 2928 native Spanish-speaking participants recruited across five Latin American countries (Argentina, Chile, Mexico, Peru, and Colombia) through the Multi-Partner Consortium to Expand Dementia Research in Latin America (ReDLat) (*29*). Of these, 1504 were HCs, 24 had MCI, 1068 had AD, 255 had nldFTD (comprising patients with corticobasal syndrome, progressive supranuclear palsy, and behavioral variant frontotemporal dementia), and 77 had ldFTD (comprising patients with the nonfluent agrammatic variant and the semantic variant, primary progressive aphasia). Overall, 67% were female, and the mean age was 65.23 years (SD = 10.84; range = 18 to 88; table S1). ReDLat uses a standardized data framework and harmonized diagnostic protocols to ensure data reliability, reproducibility, and cross-site representativeness (*1*, *5*, *17*, *42*). Participants were recruited from extensive networks including (i) memory clinics, neurology departments, and affiliated hospitals; (ii) academic collaborations with universities and research institutions; (iii) community outreach programs, engaging local communities in urban and rural areas across diverse socioeconomic backgrounds; and (iv) public health initiatives and local organizations, integrating recruitment efforts with public health campaigns. No compensation was provided to participants. Clinical and cognitive assessments across ReDLat sites are based on a standardized, harmonized, normalized, and validated batteries (*29*). Diagnoses were determined by consensus among expert health care providers at each site, based on cognitive and neurological examinations, clinical interviews, and MRI (*29*). AD diagnoses followed the clinical criteria established by the National Institute of Neurological and Communicative Disorders and Stroke and the Alzheimer’s Disease and Related Disorders Association (NINCDS-ADRDA), and clinical criteria specified for MCI, nldFTD, and ldFTD. Exclusion criteria included conditions other than AD, MCI, nldFTD, or ldFTD, as well as impairments preventing task completion, such as inability to provide consent or complete testing, low fluency, speech or language disorders, major neurological/psychiatric or cerebrovascular disease, recent substance abuse, severe sensory loss, and MRI contraindications. HCs had preserved cognition and no history of neurological or psychiatric conditions. ReDLat aligns with current guidelines to increase the representativeness of samples enrolled in AD research centers (*43*), implementing inclusive recruitment strategies, strengthened community engagement, geographic decentralization, and unified inclusion and exclusion criteria. The study protocols were approved by the institutional review boards (IRB) of each contributing institution in the ReDLat consortium (Argentina: FWA00028264; Chile: FWA00001035; Mexico: FWA00028864; Peru: FWA00001113; Colombia: FWA00010121). All participants provided written informed consent in accordance with the 2013 Declaration of Helsinki.

### Speech protocol

Speech was elicited with seven standardized tasks administered in Spanish (see detailed descriptions in text S2). A video description task required viewing a short animated film and narrating it in detail. Two 60-s phonemic fluency tasks required producing words beginning with a specific letter. Two 60-s semantic fluency tasks required naming items from a given category (namely, “animals” and “vegetables”). Last, a story retelling task, based on the Craft story, required listening to a short narrative and then recounting it on two occasions: immediately (right upon the story’s end) and with a delay (∼20 to 30 min after the story’s end).

All tasks were administered in silent, dimly illuminated rooms by expert neuropsychologists. Instructions were provided orally, and visual stimuli for the video retelling tasks were presented using 7th-generation Apple iPads. Audio files were acquired via Studio Zebra’s high-quality audio recorder and saved in .wav format (44.1 kHz sampling rate, 16-bit depth). Participants were encouraged to maintain a normal speech rate throughout the tasks. Following acquisition, files were de-identified, re-encoded, and uploaded to a secure REDCap repository. For the predictive modeling stage, we extracted a standardized speech sample per participant, aggregated from the total available recordings across tasks.

### Audio preprocessing

Audios were preprocessed through TELL v.2.0’s the automated pipeline (*26*), encompassing (i) common channel normalization, (ii) stationary noise suppression without speech distortion, (iii) participants’ speech isolation through diarization, and (iv) high-quality automated transcripts.

Common channel normalization is performed with the Sound eXchange library (https://sox.sourceforge.net/) to standardize different audio acquisition conditions. The resulting output serves as a lower bound of acceptable audio quality. Normalization consists of the following steps: conversion to .wav format, conversion to mono (single channel), encoding in GSM FULL RATE with a bit rate of 13 kbps, resampling to 16 kHz, and compression with a factor of 8 and band-pass filtering from 200 Hz to 3.4 kHz (*44*, *45*).

A spectral-gating denoiser operating on the short-time Fourier transform estimates a per-frequency noise floor and attenuates time-frequency bins below that threshold (*46*). Speech segmentation is performed with the NVIDIA NeMo toolkit, which combines MarbleNet for voice activity detection, TitaNet for speaker embeddings, and a Multi-Scale Diarization Decoder for neural diarization. MarbleNet is fine-tuned on tasks performed by the participants to reflect the interactional structure of the interviews (*26*, *27*). After diarization, participant’s segments are conserved, and examiner speech is discarded.

Diarized participant audio is transcribed locally using the pretrained Whisper Large-v3 (transformer-based ASR) model by OpenAI for Spanish transcription (*26*, *27*). Transcripts undergo a two-stage curation: (i) a human pass to correct systematic errors and remove any residual nonparticipant content and (ii) LLM-assisted quality control (QC) with Google Gemini, which is used to fSAG potential mistakes during transcription or additional interviewer speech information. Data are de-identified (coarsened dates/geo; no raw audio) and shared via encrypted, DUA-governed transfers. FAIR code and aggregates are public; subject-level data are accessible via controlled access.

### Feature extraction (age predictors)

We extracted more than 700 audio-derived features from each recording-transcript pair. Features were obtained separately for (i) the video description tasks, (ii) the two phonological-fluency tasks, (iii) the two semantic-fluency tasks, (iv) immediate story retelling, and (v) delayed story retelling. This audio-derived features represents the total set of distinct source-metric pairs computed prior to any filtering or aggregation. These raw measurements were organized into key functional domains aligning with our predictive framework: speech timing (e.g., speech rate and syllabic timing), pitch-related features (e.g., *F*_0_-based measures, harmonic-to-noise ratio, jitter, and shimmer), semantic features (concreteness and granularity), lexical features (e.g., frequency), emotional content (e.g., valence and weight of basic emotions), and verbosity (e.g., informational density and pronoun profiles). To reduce dimensionality and derive robust predictors of chronological age, we constructed category-level composite indices for seven analysis categories: speech timing, pitch, concreteness, granularity, lexical features, emotional content, and verbosity. The seven speech-feature domains were defined using a theory-informed organization of the standard feature space into interpretable functional families, with specific acoustic and language domains reflecting key aspects of healthy aging and dementia, including changes in overall processing speed (speech timing), affect (pitch and emotion), and semantic memory (semantic, lexical, and verbosity features). Within each domain, predictors were then selected empirically through recursive feature addition, allowing the final model to preserve interpretability while remaining data-driven at the feature-selection stage.

The composites were defined as follows: Speech timing combined speech rate with the inverses of the minimum and mean syllable durations, which render shorter durations as larger values; pitch combined minimum harmonics-to-noise ratio, pitch skewness, harmonic-to-noise ratio skewness, maximum shimmer, mean jitter, jitter standard deviation, and maximum jitter; concreteness combined distributional summaries of concreteness scores (median, skewness, minimum, standard deviation, and kurtosis); granularity extraction consisted of mean semantic granularity; lexical features comprised mean logarithmic lexical frequency estimated from two independent frequency resources; emotion comprised the proportion of negative sentiment and emotion scores for sadness, surprise, disgust, fear, and anger; and verbosity captured lexical microstructure and pronominal profile through token-level noun counts, content-word count per type, third-person pronoun proportion per type, noun count per type, first-person pronoun proportion per token, and content-word diversity per token. A complete inventory of features, their pipeline provenance, and implementation details is provided in text S3.

Additionally, we tested whether predictor categories could differentiate diagnostic groups after adjusting for chronological age, education, and sex. To select top-ranked linguistic and acoustic contributors, we defined seven speech-feature domains using a theory-informed partition of speech signals into components that differentially index cognitive-linguistic, lexical-semantic, motor-speech, and prosodic-timing aspects of speech production. This organization allowed us to (i) preserve interpretability by grouping mechanistically related features, (ii) limit collinearity across redundant acoustic descriptors, and (iii) enable targeted sensitivity and ablation analyses comparing acoustic-dominant versus language-dominant contributions to age prediction.

### Plasma

Plasma was processed within 2 hours of collection. Whole-blood tubes were first centrifuged at 1500*g* for 10 min at room temperature, with acceleration and deceleration set to 20%. If the layers appeared disturbed, tubes were recentrifuged for 2 min under the same conditions. Using a sterile pipette, ∼90% of the plasma was carefully transferred to a prelabeled 15-ml conical tube, avoiding the buffy coat and minimizing bubble formation. To limit cellular contamination, samples were not left standing for more than 15 min after centrifugation.

The plasma was then clarified with a second centrifugation at 1500*g* for 30 min at room temperature using the same acceleration/deceleration settings. The supernatant was transferred to a new labeled 15-ml conical tube, and 1-ml aliquots were dispensed into 1.5-ml cryovials labeled with the participant ID (PIDN) and date on the side and the sample number on the cap. Plasma samples were collected at each participating site, frozen immediately, and shipped on dry ice to our central laboratory (BrainLat) in Chile (*47*).

For biomarker measurements, frozen aliquots were thawed on wet ice and centrifuged at 2000*g* for 10 min at 4°C. Plasma p-Tau217 was quantified on the Lumipulse G600II system (Fujirebio) according to the manufacturer’s instructions. The following cartridges and calibrators were used: Lumipulse G p-Tau217 Plasma (81471 and 81472). A positive control was included in every run: Lumipulse G p-Tau217 Plasma Controls (81473). In Chile, GENE XPRESS is the distributor for the Lumipulse G600II system and cartridges.

QC was rigorously maintained throughout processing and analysis. For testing, frozen aliquots underwent a single thaw cycle prior to direct analysis. Each batch included manufacturer-provided QC materials, including calibration curves and biomarker-specific positive controls. Based on QC data, precision was consistently below a 6% coefficient of variation across all assays on the Lumipulse platform.

### Diagnostic criteria and clinical/cognitive assessments

All participants underwent diagnostic evaluations conducted by experienced behavioral neurologists and geriatricians in collaboration with neuropsychologists. Each center used a harmonized, standardized, and normalized neuropsychological test battery to assess global cognitive status, frontal functioning, social cognition, neuropsychiatric symptoms, and functional status (*47*). Clinical diagnoses followed standard procedures at each research center, as detailed previously (*48*). Participants with AD or nldFTD were included based on established criteria (*49*, *50*). The diagnostic process and clinical characterization were supported by a comprehensive set of clinical and cognitive tools tapping on functional ability, executive processing, global cognition, memory, episodic memory, and visual memory. We derived two aggregate scores from the clinical/cognitive assessment: a linguistic composite, calculated by aggregating all tasks with explicit verbal components, and a nonlinguistic composite, calculated by aggregating tasks independent of verbal processing. Within the linguistic domain, the composite combined verbal memory and naming measures: immediate verbal recall from the Craft Story test, delayed verbal recall from the Craft Story test, and the total score on the Multilingual Naming Test (MINT). Within the nonlinguistic domain, the composite aggregated visuoconstructive/visual memory and executive-attentional performance: Benson Complex Figure copy, Benson Complex Figure delayed recall, Benson Complex Figure recognition, Digit Span Forward (total and span), Digit Span Backward (total and span), and Trail Making Test Part B (set-shifting; corrected performance). Additional domain-specific composites were as follows: functional ability (Functional Activities Questionnaire total plus Activities of Daily Living Questionnaire domain scores for basic, instrumental, and advanced activities); executive (Digit Span Forward span, Digit Span Backward total and span, and Trail Making Test Part B, corrected); global cognition (Mini-Mental State Examination total); memory (Craft Story immediate and delayed verbal recall); episodic memory (Craft Story delayed recall); and visual memory (Benson Complex Figure delayed recall and recognition).

All evaluations were locally validated, contextually adapted to participants’ and considering educational level and age, as previously described (*48*). For recruitment of HC participants, a general call was made to the community through local outreach to invite interested individuals. Second, some participants voluntarily approached the recruitment sites seeking cognitive evaluation and agreed to participate in the study after meeting the inclusion criteria.

HCs had to demonstrate functional independence, as indicated by a CDR (*51*) score of 0 and an MMSE (*52*) score greater than 25. Those with limited formal education underwent confirmation of normal cognition by a neuropsychologist. Additionally, each participant was required to have a study informant (≥6 months of familiarity) knowledgeable about their daily activities and cognitive/functional status.

### Social exposome

Social exposome measures were based on an MSE (*28*). Briefly, items measuring similar facets were grouped by expert consensus into dimensions, then minimum and maximum values across 319 items, and combined as weighted averages to yield scores for education, food insecurity, financial status, assets, health care access, childhood labor, subjective socioeconomic status, childhood experiences, traumatic events, and relationships. Scores were inverted where necessary so that higher values consistently reflected more favorable social determinants of health, and subsequently min-max scaled to range between 0 and 1 (a comprehensive description of these categories is provided in text S4) to produce a score between 0 and 1. Dimensional validity was assessed via confirmatory analysis. These composite factors were then entered into a structural equation model to estimate a global latent MSE score for each participant. Prior results indicate that the dimension composite scores accurately represent the latent construct and consistently capture the underlying dimensions (*1*, *15*). The characteristics of the MSE are detailed in text S4.

### Brain clocks

We implemented a standardized, multicenter, and multimodal brain-clock pipeline (*5*–*7*, *33*) within ReDLat that yields three complementary indices of biological aging: a structural clock from T1-weighted MRI, a functional clock from resting-state fMRI, and a multimodal (combined) clock-derived by averaging the age-gap estimates from both modalities. Throughout, we adopt the BAG framework, defined as predicted minus chronological age, with negative values indicating delayed and positive values indicating accelerated brain aging. Standard, harmonized preprocessing pipelines were consistently applied across datasets (for comprehensive descriptions of these clocks, see text S5).

#### Structural clock (MRI-T1)

Structural preprocessing followed the standard voxel-based morphometry CAT12 toolbox (v2.9, https://neuro-jena.github.io/cat/) pipeline (bias-field correction, denoising, skull stripping, tissue segmentation, and MNI normalization). Gray-matter volumes were extracted per region using the Automated Anatomical Labeling (AAL116) atlas (*53*), normalized by total intracranial volume, and *z*-scored before modeling. Brain age was predicted with light gradient boosting machines (LightGBM) (*54*) under nested 10-fold cross-validation (outer folds for testing; inner folds for hyperparameter tuning).

#### Functional clock (rs-fMRI)

Rs-fMRI was preprocessed with the fMRIPrep toolbox (v22.0.2, https://fmriprep.org/) (*5*) and further denoised/smoothed and band-pass filtered (0.008 to 0.09 Hz) using the CONN22 toolbox; region of interest (ROI) time series were extracted from AAL116 (*53*). We quantified higher-order functional interactions via the organizational information (Ω, O-info) (*55*) metric (Gaussian-copula entropy estimation), which indexes redundancy- versus synergy-dominant dependencies between ROI pairs relative to the rest of the brain (*5*). These connectivity features were entered into LightGBM with nested 10-fold cross-validation to predict age; the functional BAG was defined analogously to the structural BAG.

#### Multimodal (combined) clock

We obtained a third combined BAG from the average of the structural and functional BAGs. Brain age was predicted using LightGBM. This combined clock yields a single BAG value based on structural gray-matter volume from T1-MRI and functional connectivity from rs-fMRI.

#### Harmonization and evaluation

To reduce site/scanner variability, we implemented intrasubject harmonization by min-max scaling the target BAG within scanner model (*5*, *56*), ensuring consistent ranges across scanners. We did not apply age-bias correction in the main analyses, as post hoc bias adjustment can artificially inflate performance metrics and obscure true variability (*57*). All model performance estimates were obtained within the nested cross-validation framework.

### Epigenetic clocks

Peripheral whole-blood samples were collected at each site. Leukocyte genomic DNA was extracted using the Wizard Genomic DNA Purification Kit (Promega) and quantified with fluorescence-based assays. DNA was bisulfite converted and profiled on the Infinium MethylationEPIC (850k) BeadChip following Illumina protocols (*58*, *59*).

Raw array files (IDAT format) were processed in the minfi framework using Noob or single-sample Noob background correction and dye-bias normalization. Probes that failed detection-p thresholds, probes known to be cross-reactive or affected by single-nucleotide polymorphisms, and samples failing control metrics or sex checks were removed. Where appropriate, we applied functional normalization, BMIQ to harmonize type-I and type-II probe designs, and ComBat to adjust residual batch effects. Estimated leukocyte cell-type proportions (Houseman reference-based deconvolution) were included as covariates in downstream models (*60*).

Epigenetic age (in years) was calculated using validated DNA-methylation clocks: the Hannum, Retroclock, and OMICmAge (*61*), a DNA-methylation–based estimator of telomere length. Clock values were derived with the original authors’ code and weights. We additionally referenced retroelement-based methylation clocks (*62*) (for example, ribosomal DNA–focused clocks) and multiomics clocks (for example, proteomic aging models) in sensitivity or contextual analyses where data permitted (*63*).

EAA was defined as the residual from a regression of DNA-methylation age on chronological age; positive values indicate “older-than-expected” biological age. This approach has been used extensively to capture age acceleration in clinical and population studies, and we applied it to first- and second-generation clocks [Hannum, retroelement- and multiomics-based clocks where applicable; (*10*, *64*)].

### Statistical analysis and predictor-based age estimation

We used a histogram-based gradient boosting regressor (*5*) to predict participants’ chronological age from normalized composite speech features. As predictors, we included speech timing, lexical features, emotional content, pitch, granularity, concreteness, and verbosity. These composites incorporated multiple distributional statistics for continuous variables (including mean, median, standard deviation, minimum, maximum, skewness, and kurtosis) alongside single values for ratio-based metrics. Model validation used nested 10-fold cross-validation, with 10 outer folds for performance estimation and five inner folds for hyperparameter tuning, ensuring robust estimation and mitigating overfitting (*5*). Model performance was assessed using the *R*^2^, MDE, MAE, and RMSE (*9*). Additionally, Cohen’s *f*^2^ was computed to quantify effect sizes (*65*). Feature importance was determined via MDI (*66*), averaged across outer folds. To optimize the model’s hyperparameters, grid search was used.

We applied a synthetic minority over-sampling technique for regression with Gaussian noise (SMOGN) (*67*) to perform data augmentation exclusively within the HC group. Augmented samples were used only in the age regression model; no synthetic data were added to patient groups, and evaluation relied on the original, non-augmented data. Results without any data augmentation are reported in text S1.

### SAG estimation

SAGs were computed as predicted age minus chronological age and are interpreted here as cross-sectional deviations from expected age-related speech profiles (Fig. 1D), with positive values (SAG > 0) indicating relatively older-appearing speech and negative values indicating relatively younger-appearing or preserved speech (SAG < 0) (*68*). To account for regression-to-the-mean bias (*9*), we regressed out chronological age from the estimated SAGs and used the residuals for SAG correction. Regression coefficients were obtained from the training data and subsequently applied to the test data. Statistical differences in SAGs were compared across diagnostic groups. Permutation tests with 10,000 iterations ensured robustness to non-normality. Bonferroni correction addressed multiple comparisons. Effect sizes were calculated using Cliff’s delta.

### SAG associations across phenotypic, biological, social, and aging-clock measures

We examined the associations between the SAG and a comprehensive set of phenotypic, biological, social, and multimodal aging-clock measures using linear regression models. The mathematical representation of the linear regression model is

$$Y=B_{0}+B_{1}x+\epsilon$$

where Y is the outcome variable, $B_{0}$ is the intercept, $B_{1}$ is the slope associated with the independent variable X, and ϵ represents the error term. The coefficient $B_{1}$ is determined using the least squares method, which aims to minimize the sum of the squared differences (errors) between the observed values (actual values) and the values predicted by the model. The formulas to calculate the coefficients are

$$B={\left(X^{T}X\right)}^{-1}X^{T}Y$$

where $X^{T}$ is the transpose of matrix of vector *X*.

### Sensitivity analyses

We conducted multiple sensitivity analyses to further evaluate the robustness of the findings. All primary analyses were repeated without data augmentation. In addition, we implemented a LOCO cross-validation strategy by iteratively excluding each country from the training set and evaluating model performance on the held-out country. Associations between clinical and cognitive measures were examined using linear regression models without applying any a priori grouping. Given the known influence of biological sex on vocal features, we conducted a sensitivity analysis including sex as a covariate in the age-prediction model. Additionally, we performed matching by age, sex, and education. Matching analyses were conducted in R version 4.4.2 (2024-10-31). Propensity score matching was implemented using the MatchIt package, and covariate balance was assessed with the cobalt package. Nearest-neighbor matching was used to balance key covariates, including age, years of education, and sex. Covariate balance was evaluated using numerical summaries and visual diagnostics. After matching, group differences were examined statistically.
