# Supplementary material for: Speech clocks decode dementia phenotypes, social exposome, and biological aging
Source: Sci Adv. 2026 Sep 30;12(40):eaef9864. doi: 10.1126/sciadv.aef9864 (PMC13626050; doi:10.1126/sciadv.aef9864)
Supplement: Supplementary file 1 [file sciadv.aef9864_sm.pdf]

Science Advances
AAAS

Supplementary Materials for

Speech clocks decode dementia phenotypes, social exposome, and biological aging

Hernan Hernandez *et al.*

Corresponding author: Adolfo M. García, adolfo.garcia@gbhi.org; Agustin Ibanez, agustin.ibanez@gbhi.org

Sci. Adv. **12**, eaef9864 (2026)
DOI: 10.1126/sciadv.aef9864

This PDF file includes:

Supplementary Text
Figs. S1 and S2
Tables S1 to S10

Supplementary Text

Supplementary Information 1. Supplementary Figures and Data

Fig. S1.
Capturing diagnostic categories via SAGs using a leave-one-country-out (LOCO) approach without data augmentation.

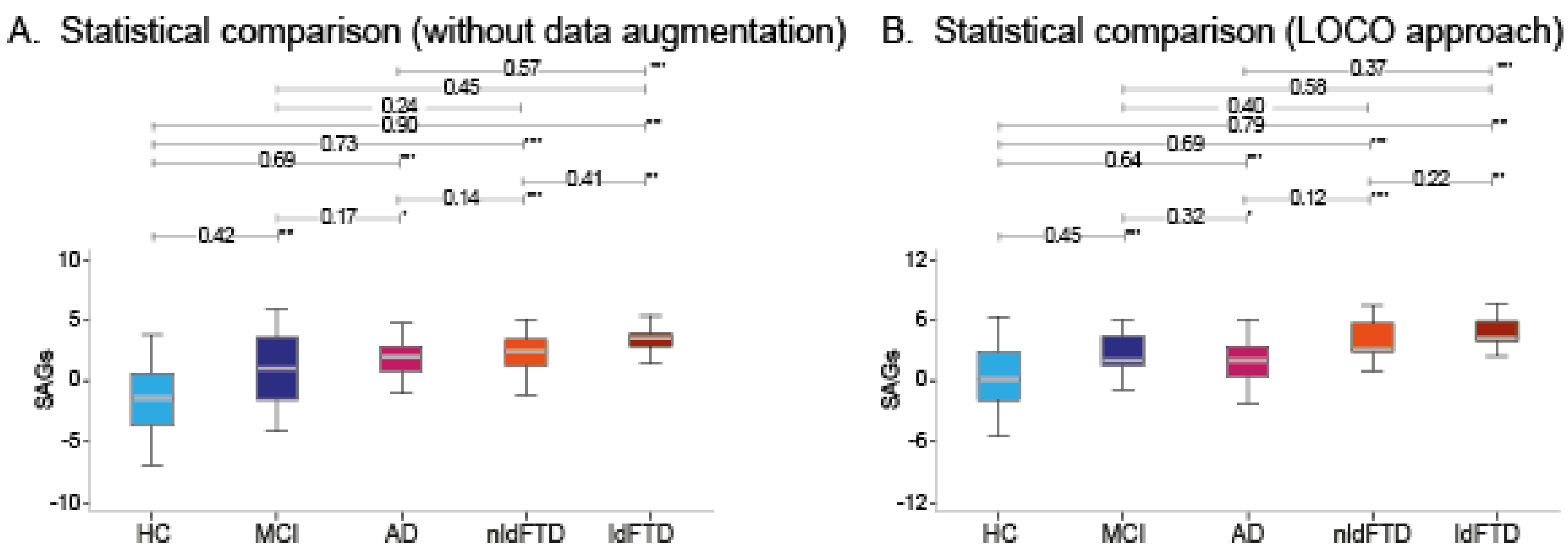

We analyzed whether SAGs differentiate between HCs and patient groups without data augmentation and using a leave-one-country-out (LOCO) approach. HCs had lower SAGs than every patient group (No-Augmentation: Cliff's delta (δd) = 0.42–0.90; LOCO: Cliff's delta (δd) = 0.32–0.69; all $P < 0.01$), evidencing a more pronounced accelerated aging in patients with neurodegenerative diseases. Within patient cohorts, ldFTD showed the greatest SAGs, surpassing AD (No-Augmentation: Cliff's delta (δd) = 0.57, $P < 1 \times 10^{-15}$; LOCO: Cliff's delta (δd) = 0.37, $P < 1 \times 10^{-15}$) and nldFTD (No-Augmentation: Cliff's delta (δd) = 0.41, $P < 1 \times 10^{-15}$; LOCO: Cliff's delta (δd) = 0.22, $P = 0.0051$). nldFTD involved greater SAGs than AD (No-Augmentation:

Cliff's delta (δd) = 0.14, P = 0,0037; LOCO: Cliff's delta (δd) = 0.12, P = 0,0065). Extended data are detailed below:

Table Fig S1-A. Statistical comparisons between diagnostic groups (No-Augmentation). Cliff's Delta effect sizes and associated p-values for comparisons between diagnostic groups without data augmentation.

**Group1**	**Group2**	**CliffsDelta**	**p_value**
**HC**	**MCI**	**0,42**	**0,0001**
**HC**	**AD**	**0,69**	**0**
**HC**	**nldFTD**	**0,73**	**0**
**HC**	**ldFTD**	**0,9**	**0**
**MCI**	**AD**	**0,17**	**0,0047**
**MCI**	**nldFTD**	**0,24**	**0,0029**
**MCI**	**ldFTD**	**0,44**	**0**
**AD**	**nldFTD**	**0,14**	**0,0037**
**AD**	**ldFTD**	**0,57**	**0**
**nldFTD**	**ldFTD**	**0,41**	**0**

Table Fig S1-B Statistical comparisons between diagnostic groups (Leave-one-country-out (LOCO). Cliff's Delta effect sizes and associated p-values for comparisons between diagnostic groups with LOCO approach.

**Group1**	**Group2**	**CliffsDelta**	**p_value**
**HC**	**MCI**	**0,42**	**0,0011**
**HC**	**AD**	**0,64**	**0**
**HC**	**nldFTD**	**0,68**	**0**
**HC**	**ldFTD**	**0,79**	**0**
**MCI**	**AD**	**0,32**	**0,0007**
**MCI**	**nldFTD**	**0,4**	**0,0003**
**MCI**	**ldFTD**	**0,58**	**0**
**AD**	**nldFTD**	**0,12**	**0,0065**
**AD**	**ldFTD**	**0,36**	**0**
**nldFTD**	**ldFTD**	**0,22**	**0,0051**

Figure S2.
Association between speech age gaps (SAGs) and network-based brain age gaps (BAGs). Scatter plots show the relationship between SAGs and network-derived BAGs across overall BAG and linguistic **(a),** and non-linguistic **(b)** brain networks. Each point represents an individual, and solid lines indicate linear fits for each network. Line opacity reflects statistical significance, with transparent lines indicating non-significant associations. **(a)** Linguistic networks include a Global Language Network (GLN), a Verbal Production Network, and whole-brain clocks for comparison. **(b)** Non-linguistic networks include the Default Mode Network (DMN), Executive Network (EN), Motor Network (MN), Salience Network (SN), and Visual Network (VN).

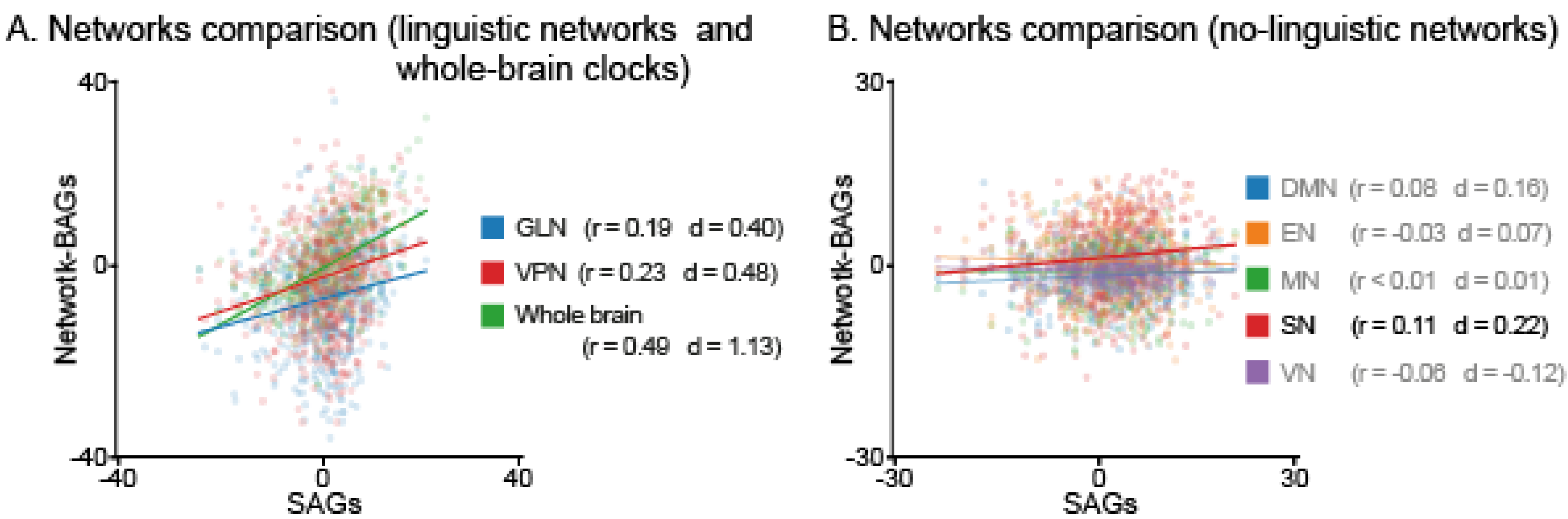

Table S1.
Age distribution and sex composition of the sample by country. The table reports mean age, standard deviation, minimum and maximum age, total sample size (n_total), and number of female participants (n_female) for each country.

Country	mean_age	sd_age	min_age	max_age	n_total	n_female
Argentina	63,52	13,11	18	80	195	125
Chile	65,90	9,97	40	81	299	209
Colombia	65,53	11,68	19	88	1127	715
Mexico	63,55	11,33	23	80	509	363
Peru	66,08	8,66	40	82	798	562

Table S2. Statistics for within-group comparison of SAGs. Cliff's Delta effect sizes and associated p-values for all pairwise comparisons of SAGs across diagnostic groups.

Group1	Group2	CliffsDelta	p_value
HC	MCI	0.35	0

HC	AD	0.33	0
HC	nldFTD	0.48	0
HC	ldFTD	0.76	0
MCI	AD	0.1	0.0108
MCI	nldFTD	0	0.7337
MCI	ldFTD	0.23	0.0734
AD	nldFTD	0.23	0
AD	ldFTD	0.65	0
nldFTD	ldFTD	0.35	0.0002

Table S3. Correlations between SAGs and plasma p-Tau217 across diagnostic groups. The table reports Pearson's r, 95% confidence intervals, and p-values.

	**Group**	**N**	**R**	**r_CI_low**	**r_CI_high**	**p_value**
p-tau217	HC	181	-0,035557327	-0,180479061	0,110875462	0,634636593
p-tau217	AD	185	0,18105904	0,037777224	0,317046038	0,013647991
p-tau217	nldFTD	79	0,173155905	-0,049863603	0,37972783	0,126993673
p-tau217	ldFTD	22	-0,273117359	-0,622989452	0,167814715	0,218770168

Table S4. Correlations between SAGs and clinical/cognitive performance across groups. The table reports Pearson's r, 95% confidence intervals and p-values.

Composite score	Group	N	r	r_CI_low	r_CI_high	p_value
Functional ability	HC	1493	0,060249028	0,00954621	0,110642839	0,01990419
Functional ability	MCI	24	0,296564546	-0,1213496	0,625170171	0,159369361
Functional ability	AD	1059	-0,128498735	-0,187289647	-0,068790539	2,73955E-05
Functional ability	nldFTD	252	-0,140281868	-0,259358725	-0,017003781	0,025956835
Functional ability	ldFTD	77	-0,12657981	-0,340893892	0,100240974	0,272647862
Executive	HC	1494	-0,222857752	-0,270515335	-0,17411058	2,86682E-18
Executive	MCI	24	0,011536576	-0,393692305	0,413011208	0,957332006
Executive	AD	1060	-0,237286403	-0,29330801	-0,179640771	4,93959E-15
Executive	nldFTD	254	-0,30796233	-0,41530459	-0,19216175	5,55439E-07
Executive	ldFTD	75	-0,178769998	-0,389911583	0,050229872	0,124886762
Global_Cognition	HC	1499	-0,235311271	-0,282575037	-0,186907735	2,63471E-20
Global_Cognition	MCI	24	0,06359824	-0,348745652	0,45531373	0,767816069
Global_Cognition	AD	1066	-0,233811479	-0,289785783	-0,176243194	1,05596E-14
Global_Cognition	nldFTD	255	-0,312636421	-0,419372938	-0,197374021	3,46551E-07
Global_Cognition	ldFTD	77	-0,281857835	-0,475799364	-0,061779132	0,01301308
Memory	HC	1491	-0,303293003	-0,348690247	-0,256475937	4,2697E-33
Memory	MCI	24	-0,238756519	-0,585738635	0,182187305	0,261196494
Memory	AD	1058	-0,345235895	-0,397239665	-0,291022046	5,58849E-31
Memory	nldFTD	250	-0,420688385	-0,517733064	-0,312955645	3,83311E-12
Memory	ldFTD	75	-0,368432415	-0,549449258	-0,154379827	0,001144562
Episodic memory	HC	1490	-0,28232828	-0,328402864	-0,234913289	1,05135E-28
Episodic memory	MCI	24	-0,329584007	-0,646964745	0,085131154	0,115778436
Episodic memory	AD	1054	-0,331238111	-0,383942234	-0,276382655	2,08327E-28
Episodic memory	nldFTD	249	-0,398776955	-0,498386042	-0,288776588	6,40434E-11
Episodic memory	ldFTD	75	-0,386195144	-0,563743951	-0,174531235	0,000621237
Visual memory	HC	1493	-0,057748316	-0,108163427	-0,007036935	0,025657876
Visual memory	MCI	24	-0,03622068	-0,433286509	0,372620501	0,866562503
Visual memory	AD	1059	-0,172318577	-0,23016997	-0,113253523	1,66839E-08
Visual memory	nldFTD	252	-0,202391084	-0,318010477	-0,080840049	0,001236251
Visual memory	ldFTD	77	0,031617002	-0,193733764	0,253798549	0,78487749

Table S5. Correlations between SAGs and linguistic and non-linguistic composite scores groups. The table reports Pearson's r, 95% confidence intervals and p-values.

Composite score	Group	N	r	r_CI_low	r_CI_high	p_value
Linguistic	HC	1494	-0,314063267	-0,359059281	-0,267610685	1,48252E-35
Linguistic	MCI	24	-0,203084948	-0,56055857	0,218186422	0,341215679
Linguistic	AD	1063	-0,27292886	-0,327678704	-0,216352096	1,29759E-19
Linguistic	nldFTD	253	-0,402541196	-0,500997361	-0,29379874	2,83762E-11
Linguistic	ldFTD	77	-0,355071205	-0,536391649	-0,14241871	0,001531187
Non_Linguistic	HC	1494	-0,177838249	-0,226510347	-0,128280207	4,41054E-12
Non_Linguistic	MCI	24	-0,015894072	-0,416619577	0,390003188	0,941239226
Non_Linguistic	AD	1063	-0,286460889	-0,340719504	-0,230300405	1,58665E-21

Non_Linguistic	nldFTD	254	-0,309616992	-0,416817032	-0,193922654	4,79358E-07
Non_Linguistic	ldFTD	77	-0,097374328	-0,31449411	0,129427286	0,399516491

Table S6. Correlations between SAGs and individual clinical/cognitive performance across groups. The table reports Pearson's r, 95% confidence intervals and p-values.

Clinical/Cognitive	Group	N	R	r_CI_low	r_CI_high	p_value
Benson Complex Figure copy	HC	1493	-0.10	-0.15	-0.05	0.000154
Benson Complex Figure copy	MCI	24	0.12	-0.30	0.50	0.5885
Benson Complex Figure copy	AD	1058	-0.26	-0.31	-0.20	3.05E-17
Benson Complex Figure copy	nldFTD	252	-0.24	-0.36	-0.12	9.53E-05
Benson Complex Figure copy	ldFTD	77	-0.10	-0.32	0.13	0.384484
Benson Complex Figure delayed recall	HC	1493	-0.07	-0.12	-0.02	0.006149
Benson Complex Figure delayed recall	MCI	24	-0.38	-0.68	0.02	0.064677
Benson Complex Figure delayed recall	AD	1049	-0.28	-0.33	-0.22	2.42E-20
Benson Complex Figure delayed recall	nldFTD	249	-0.29	-0.40	-0.18	2.56E-06
Benson Complex Figure delayed recall	ldFTD	77	0.02	-0.21	0.24	0.869866
Benson Complex Figure recognition	HC	1492	-0.03	-0.09	0.02	0.182296
Benson Complex Figure recognition	MCI	24	0.11	-0.30	0.49	0.600934
Benson Complex Figure recognition	AD	1059	-0.09	-0.15	-0.03	0.0036
Benson Complex Figure recognition	nldFTD	252	-0.12	-0.24	0.01	0.062624
Benson Complex Figure recognition	ldFTD	77	0.03	-0.19	0.25	0.78537
Verbal recall from the Craft Story test	HC	1491	-0.31	-0.35	-0.26	5.5E-34
Verbal recall from the Craft Story test	MCI	24	-0.13	-0.51	0.28	0.529908
Verbal recall from the Craft Story test	AD	1058	-0.31	-0.37	-0.26	2.28E-25
Verbal recall from the Craft Story test	nldFTD	250	-0.41	-0.51	-0.30	1.58E-11
Verbal recall from the Craft Story test	ldFTD	75	-0.33	-0.52	-0.11	0.003828
Delayed verbal recall from the Craft Story test	HC	1490	-0.28	-0.33	-0.23	1.05E-28
Delayed verbal recall from the Craft Story test	MCI	24	-0.33	-0.65	0.09	0.115778
Delayed verbal recall from the Craft Story test	AD	1054	-0.33	-0.38	-0.28	2.08E-28
Delayed verbal recall from the Craft Story test	nldFTD	249	-0.40	-0.50	-0.29	6.4E-11
Delayed verbal recall from the Craft Story test	ldFTD	75	-0.39	-0.56	-0.17	0.000621
Digit Span Backward	HC	1485	-0.17	-0.22	-0.12	7.78E-11
Digit Span Backward	MCI	24	0.03	-0.38	0.43	0.8999
Digit Span Backward	AD	1053	-0.18	-0.24	-0.12	3.59E-09
Digit Span Backward	nldFTD	243	-0.27	-0.38	-0.14	2.8E-05
Digit Span Backward	ldFTD	75	-0.20	-0.41	0.03	0.087609
Digit Span Backward total	HC	1485	-0.18	-0.23	-0.13	2.55E-12
Digit Span Backward total	MCI	24	-0.09	-0.48	0.33	0.677334
Digit Span Backward total	AD	1052	-0.19	-0.25	-0.13	7.98E-10
Digit Span Backward total	nldFTD	241	-0.26	-0.38	-0.14	3.66E-05
Digit Span Backward total	ldFTD	75	-0.22	-0.42	0.01	0.062236
Digit Span Forward	HC	1494	-0.16	-0.21	-0.11	1.34E-09
Digit Span Forward	MCI	24	-0.01	-0.41	0.39	0.960625
Digit Span Forward	AD	1059	-0.14	-0.20	-0.08	5.01E-06

Digit Span Forward	nldFTD	253	-0.26	-0.37	-0.14	2.45E-05
Digit Span Forward	ldFTD	75	-0.09	-0.31	0.14	0.422101
Digit Span Forward total	HC	1494	-0.16	-0.21	-0.11	7.74E-10
Digit Span Forward total	MCI	24	-0.12	-0.50	0.30	0.576413
Digit Span Forward total	AD	1059	-0.16	-0.22	-0.10	1.02E-07
Digit Span Forward total	nldFTD	253	-0.20	-0.32	-0.08	0.00111
Digit Span Forward total	ldFTD	75	-0.10	-0.32	0.13	0.409903
Multilingual Naming Test	HC	1494	-0.20	-0.25	-0.15	1.76E-15
Multilingual Naming Test	MCI	24	-0.03	-0.43	0.37	0.87268
Multilingual Naming Test	AD	1059	-0.12	-0.18	-0.06	7.4E-05
Multilingual Naming Test	nldFTD	249	-0.26	-0.38	-0.14	2.57E-05
Multilingual Naming Test	ldFTD	74	-0.25	-0.45	-0.02	0.032531
Trail Making Test Part B	HC	1387	-0.07	-0.12	-0.01	0.014864
Trail Making Test Part B	MCI	21	-0.27	-0.63	0.19	0.243132
Trail Making Test Part B	AD	760	-0.16	-0.23	-0.09	7.41E-06
Trail Making Test Part B	nldFTD	181	-0.16	-0.30	-0.01	0.036166
Trail Making Test Part B	ldFTD	52	0.01	-0.27	0.28	0.957012
Mini-Mental State Examination total	HC	1499	-0.24	-0.28	-0.19	2.63E-20
Mini-Mental State Examination total	MCI	24	0.06	-0.35	0.46	0.767816
Mini-Mental State Examination total	AD	1066	-0.23	-0.29	-0.18	1.06E-14
Mini-Mental State Examination total	nldFTD	255	-0.31	-0.42	-0.20	3.47E-07
Mini-Mental State Examination total	ldFTD	77	-0.28	-0.48	-0.06	0.013013
ADLQ advanced activities	HC	1491	0.09	0.04	0.14	0.000535
ADLQ advanced activities	MCI	24	0.37	-0.04	0.67	0.072706
ADLQ advanced activities	AD	1053	-0.11	-0.17	-0.05	0.000249
ADLQ advanced activities	nldFTD	251	-0.11	-0.23	0.01	0.083754
ADLQ advanced activities	ldFTD	76	-0.02	-0.24	0.21	0.87522
ADLQ basic activities	HC	1491	0.02	-0.03	0.07	0.536634
ADLQ basic activities	MCI	24	0.06	-0.35	0.45	0.793076
ADLQ basic activities	AD	1053	-0.05	-0.11	0.01	0.090171
ADLQ basic activities	nldFTD	251	-0.08	-0.20	0.04	0.200624
ADLQ basic activities	ldFTD	76	-0.19	-0.40	0.04	0.10592
ADLQ instrumental activities	HC	1491	0.04	-0.01	0.09	0.166052
ADLQ instrumental activities	MCI	24	0.29	-0.13	0.62	0.170069
ADLQ instrumental activities	AD	1053	-0.13	-0.19	-0.07	2.47E-05
ADLQ instrumental activities	nldFTD	251	-0.15	-0.27	-0.02	0.019545
ADLQ instrumental activities	ldFTD	76	-0.09	-0.31	0.13	0.422609
Functional Activities Questionnaire total	HC	1473	-0.06	-0.11	-0.01	0.021099
Functional Activities Questionnaire total	MCI	24	0.08	-0.33	0.47	0.695621
Functional Activities Questionnaire total	AD	1041	-0.10	-0.16	-0.04	0.000722
Functional Activities Questionnaire total	nldFTD	250	-0.15	-0.27	-0.02	0.020215

Functional Activities Questionnaire total	ldFTD	77	-0.24	-0.44	-0.01	0.037792

Table S7. Correlations between SAGs and the Multimodal Social Exposome (MSE) factor across groups. The table reports Pearson's r, 95% confidence intervals and p-values.

	Group	N	r	r_CI_low	r_CI_high	p_value
MSE_factor	HC	981	-0,282549423	-0,339142498	-0,223918614	1,83163E-19
MSE_factor	MCI	9	-0,111085737	-0,721946258	0,597085635	0,776012237
MSE_factor	AD	691	-0,137950609	-0,210370288	-0,064025196	0,000275694
MSE_factor	nldFTD	151	-0,086101343	-0,242494824	0,074653831	0,293170202
MSE_factor	ldFTD	48	-0,046259294	-0,326107675	0,241043651	0,754878766

Table S8. Correlations between SAGs and brain age gaps (BAGs) derived from functional, structural, and combined MRI clocks across groups. The table reports Pearson's r, 95% confidence intervals and p-values.

Clock	Group	nclean	r	r_CI_low	r_CI_high	p_value
BAG_functional	HC	158	0,44412426	0,309442448	0,561338184	5,05875E-09
BAG_functional	AD	285	0,571444067	0,487633118	0,644818769	4,17794E-26
BAG_functional	nldFTD	52	0,370611575	0,108705758	0,584405147	0,006838561
BAG_functional	ldFTD	26	0,606491463	0,286429054	0,804779953	0,001021455
BAG_structural	HC	158	0,385035245	0,243536211	0,510485419	5,87308E-07
BAG_structural	AD	285	0,402421364	0,300273959	0,495443396	1,60929E-12
BAG_structural	nldFTD	52	0,425875265	0,173091952	0,626018748	0,001645396
BAG_structural	ldFTD	26	0,435943197	0,058463627	0,704355279	0,025996365
BAG_combined	HC	158	0,473112167	0,342254863	0,585966066	3,44401E-10
BAG_combined	AD	285	0,587212428	0,505514871	0,658474114	8,30223E-28
BAG_combined	nldFTD	52	0,430753415	0,178881055	0,629638053	0,001433905
BAG_combined	ldFTD	26	0,625093235	0,313697116	0,815087226	0,000639486

Table S9. Correlations between SAGs and epigenetic clocks derived from the Hannum, Retroclock, and OMICmAge clocks across groups. The table reports Pearson's r, 95% confidence intervals and p-values.

Clock	Group	N	r	r_CI_low	r_CI_high	p_value
Hannum	HC	147	0,315625582	0,16201066	0,454304948	9,85613E-05

Hannum	AD	196	0,196357292	0,057795151	0,327497232	0,005809876
Hannum	nldFTD	44	0,236176492	-0,065281142	0,498129904	0,122723153
Hannum	ldFTD	12	0,414173104	-0,209533496	0,798318672	0,180719888
Retroclock	HC	147	0,201719642	0,041170874	0,352113926	0,01428234
Retroclock	AD	196	0,364574591	0,236506487	0,480191349	1,49372E-07
Retroclock	nldFTD	44	0,236736092	-0,064690904	0,498575435	0,121813006
Retroclock	ldFTD	12	0,579210322	0,007952042	0,865433436	0,048433817
OMICmAge	HC	147	0,323113633	0,170119273	0,46089741	6,5598E-05
OMICmAge	AD	196	0,291808683	0,158122892	0,414989215	3,32661E-05
OMICmAge	nldFTD	44	0,317712309	0,023001498	0,561619315	0,03558939
OMICmAge	ldFTD	12	0,603245912	0,044882939	0,874419999	0,037838961

Table S10. ANCOVA models. Results across three progressively stringent models assessing the influence of individual covariates on the SAGs.

Model 1: Base Model

Predictor	*sum of squares*	*df*	*F*	PR(>*F*)
Clinical diagnosis	24813.854860	4.0	172.281203	1.615229e-132
Sex	699.163782	1.0	19.417020	1.088833e-05
Country	4229.235948	4.0	29.363348	5.729852e-24
Education	6996.856675	1.0	194.315134	8.508403e-43
Age	44518.951772	1.0	1236.370342	7.252636e-226
Residual	104278.532034	2896.0	NaN	NaN

Model 2: Base + Comorbidity

Predictor	*sum of squares*	*df*	*F*	PR(>*F*)
Clinical diagnosis	24859.274760	4.0	172.667740	8.852781e-133
Sex	702.928781	1.0	19.529632	1.026925e-05
Country	3899.686815	4.0	27.086474	4.253604e-22
Education	6737.347643	1.0	187.185283	2.459111e-41
Age	44320.691609	1.0	1231.371993	4.431779e-225
Comorbidity	114.406194	1.0	3.178574	7.471462e-02
Residual	104163.552724	2894.0	NaN	NaN

Model 3: Base + Comorbidity + Language

Predictor	*sum of squares*	*df*	*F*	PR(>*F*)
Clinical diagnosis	24299.800455	4.0	168.897595	4.477345e-130
Sex	718.067918	1.0	19.963941	8.195910e-06
Country	3831.404997	4.0	26.630469	1.012940e-21
Education	6360.110527	1.0	176.825711	3.342373e-39
Age	43936.436448	1.0	1221.534055	2.076590e-223
Number of languages	10.917151	1.0	0.303522	5.817256e-01
Comorbidity	111.348977	1.0	3.095758	7.860276e-02
Residual	103588.546239	2880.0	NaN	NaN

__Table S10.__ Type II sum of squares; df = degrees of freedom; F = F-statistic; PR(>F) = p-value. NaN values indicate the residual term where F and p-values are not applicable.

Supplementary Information 2. Task used to elicit speech samples

Video description task

In the video description task, participants viewed one of two video narratives, prompted with this instruction: "Watch the following video. Then describe the story of the video. Mention all the details you can." One video showed a fish trying and failing to avoid being cooked; the other depicted a dog that first resented a bird for stealing bait worms but the realized it did so to feed its family. Immediately after viewing their assigned video, participants produced an unprompted free narrative. Examiners minimized feedback and used only content-free encouragement if the participant paused (e.g., "keep going"), avoiding specific cues or corrections. This task elicits a partly structured, predictable speech sample, as descriptions are expected to include key informational/semantic elements such as subjects, objects, actions, and places depicted in the videos. Typical administration time in HCs is ~2–5 minutes.

Phonemic verbal fluency

Participants completed two 60-s phonemic fluency tasks each requiring rapid generation of unique Spanish words beginning with the designated letter (P and M). Standard rules applied: proper names, numbers, and repetitions were not allowed; morphological variants of the same root were not counted as additional responses; and intrusions (words not beginning with the target letter) were excluded. One neutral prompt (e.g., "keep going" or "what other words beginning with [letter] can you think of?") was permitted after 15 s of silence. The outcome for each trial was the count of valid, unique responses produced within 60 s.

Semantic category fluency

Participants completed two 60-s semantic fluency trials, each requiring rapid generation of unique items from a designated category (namely, 'animals' and 'vegetables'). As per standard guidance, one content-free prompt was permitted after 15 s without a response; repetitions and out-of-category items were excluded from scoring. The outcome for each trial was the count of valid, unique category members produced within 60 s.

Immediate and delayed story retelling

Story retelling was assessed with Craft Story 21 from the NACC UDS v3 battery. For immediate retelling, the examiner read the Craft Story 21 passage once, aloud, at a normal pace with clear articulation and adequate volume, without repetitions. Immediately after presentation, participants produced a free recall from memory. Examiners followed the standardized instructions (fixed wording for read-aloud and recall prompts) and, upon completing the immediate trial, delivered the future-recall cue and documented the exact time to compute the delay interval. Administration and scoring adhered to UDS v3 worksheets/instructions, which specify both verbatim (maximum 44 units) and paraphrase (maximum 25 units) scoring, with explicit criteria for allowable paraphrases and non-creditable intrusions or repetitions.

For delayed retelling, the story was recalled again ~20 minutes after CraftIm. Per UDS v3 guidelines, the neuropsychological battery was administered in the prescribed order to standardize

delay intervals across sites; if the target delay had not elapsed, no additional cognitive tests were inserted solely to fill the interval. If needed, a single neutral cue (e.g., “It was a story about a boy …”) was permitted. Delayed recall used the same verbatim/paraphrase scoring framework as the immediate condition, and the actual elapsed delay (minutes) was recorded on the worksheet.

Supplementary Information 3. Acoustic and linguistic features.

Speech timing. This category characterizes the temporal fluency and processing speed of speech using automated timing extraction algorithms from the TELL v.2.0 pipeline(24). Key metrics included speech rate (approximated as syllables per second) and syllabic timing indices. To ensure consistent directionality across the predictive model, where higher values represent preserved function, we computed inverse-duration metrics (e.g., the inverse of mean and minimum syllable durations). Consequently, higher values in this category correspond to shorter syllable durations and faster articulation speeds, reflecting preserved motor-speech planning. Segmentation of speech and silence was performed using a voice activity detector implemented in pyannote v1.1.00 (64). The detector is based on a neural architecture combining Sinc convolutional filters with a long short-term memory (LSTM) network, producing probabilistic estimates of speech presence for each audio segment. These probabilities were thresholded to generate binary speech–silence labels, incorporating onset and offset criteria as well as minimum segment duration constraints. Speech rate was derived by dividing the number of transcribed words by the total speech duration in minutes after excluding silent segments. Silence duration was quantified as the mean length of silent intervals, while silences per word were computed as the number of silence segments normalized by the total number of words.

Pitch. This category characterizes the intonation and tonal height of the voice based on the fundamental frequency (F0), which reflects the rate of vocal fold vibration during phonation. F0 trajectories were extracted from voiced segments using autocorrelation methods. To capture the distributional properties of prosody beyond simple averages, we computed pitch skewness. This metric quantifies the asymmetry of the F0 distribution over time, serving as an index of the speaker's tendency to deviate towards the lower or higher extremes of their vocal range relative to the central tendency. Pitch estimation was performed using openSMILE and the eGeMAPSv02 feature set, relying on sub-harmonic sampling combined with Viterbi smoothing(65). The resulting pitch contour was sampled at 100 Hz and restricted to a frequency range between 30 and 500 Hz. Pitch level and pitch variability were operationalized as the mean and standard deviation (Hz) computed over all voiced segments. The full pitch time series was preserved within the processing pipeline to enable further analyses if required.

Concreteness. Concreteness quantifies the extent to which a word refers to a perceptible, material entity that can be experienced through the senses. Consistent with prior studies (66, 67), word concreteness was automatically derived from the EsPal database(68), which provides ratings on a 1–7 scale from highly abstract to highly concrete. To maximize the explanatory power of our multivariate approach, each feature was summarized using seven distributional descriptors per task: mean, median, standard deviation, minimum, maximum, skewness, and kurtosis(66).

Granularity. This category quantifies the level of semantic specificity in lexical selection, distinguishing between generic terms (e.g., 'animal') and highly specific concepts (e.g., 'poodle').

Following the TELL v.2.0 pipeline(24), granularity was computed using ontology-based algorithms mapped to WordNet hierarchies. Specifically, we calculated the hypernym depth for each content word, defined as the distance (number of nodes) from the root of the semantic tree to the target lexical item. These item-level indices were averaged to yield a sample-level estimate, where higher values indicate a deeper traversal of the semantic network and, consequently, finer-grained lexical specificity. We estimated word granularity using established methods (66, 69). WordNet was accessed through Python's NLTK toolkit(70), which provides a hierarchical graph of noun synsets structured from the least specific hypernym ("entity") toward increasingly fine-grained concepts (e.g., "animal," "dog," "bulldog"). Granularity was operationalized as the shortest path length between each produced word and the root node "entity," with smaller distances reflecting lower semantic specificity.

Lexical features. These metrics quantify sublexical and usage-related properties of words. As in previous works,(66, 67) we used the EsPal database(68) to automatically derive each word's length (number of phonemes), phonological neighborhood (number of words obtained upon substituting, adding, or omitting a phoneme), and frequency (logarithmic frequency per million). To maximally exploit our multivariate approach, each property was analyzed in terms of seven distributional features(66) per task, namely: mean, median, standard deviation, minimum, maximum, skewness, and kurtosis.

Emotional content. To capture the affective dimension of speech, we employed state-of-the-art Transformer-based language models optimized for Spanish text classification, as implemented in the TELL v.2.0 pipeline(24). This approach leverages contextual embeddings to infer emotional states beyond simple keyword matching. Linguistic emotionality was quantified using PySentimiento(71), a multilingual Python library grounded in RoBERTa(72) and optimized for emotion assessment tasks(71). For each transcription, overall emotional valence was derived from the distribution of positive, negative, and neutral emotional expressions(71). Furthermore, leveraging a BETO model trained on emotional narratives from the EmoEvent corpus, the tool computes the relative weight of six core emotions (joy, sadness, anger, fear, disgust, and surprise) with respect to global emotionality, thereby characterizing participants' affective reactivity. In total, six emotional features were extracted per participant.

Verbosity. This category characterizes the informational structure and referential style of the discourse using automated Part-of-Speech (POS) tagging as implemented in the TELL v.2.0 pipeline(24). Rather than simple word counts, we computed the density of specific grammatical categories to capture the balance between content-heavy and referential speech. Metrics included the normalized frequency of content words (nouns, verbs, adjectives) and function words (specifically first- and third-person pronouns). These counts were normalized by the total number of words (per token) or unique word forms (per type) to control for varying speech duration and vocabulary size, yielding robust indices of semantic density. Words were automatically assigned part-of-speech labels using BETO's token_classification function(73), a BERT-based language model trained exclusively on Spanish corpora from Wikipedia and the OPUS Project(74). BETO's training data cover several Spanish dialects, including the Rioplatense variant spoken by all participants, and the model has been successfully applied in Argentinean samples(75-77). According to the Spanish Universal Dependencies Treebank (v1.4)(78), BETO assigns tokens to 17 grammatical categories. Focusing on content words, we computed the proportion of adjectives,

adverbs, nouns (including proper nouns), and verbs relative to total word count(79, 80). Manual inspection of tagging quality on five random retellings per group resulted in 96.16% accuracy, in line with previous estimates(73).

Supplementary Information 4. A multidimensional social exposome (MSE)

MSE questionnaire
The MSE questionnaire was co-designed by the ReDLat consortium and the UCSF-MAC, following the NIA Health Disparities Research Framework and the Institute of Medicine reports and Matching guidelines to increase sample representativeness in Alzheimer's disease research(36) and recommended practices for cross-national comparisons(81).

Dimensions
The MSE questionnaire was created by different experts working with Latino participants from Mexico, Central America, and South America. It was co-designed by the ReDLat consortium and the UCSF-MAC, following the NIA Health Disparities Research Framework and the Institute of Medicine reports. The questionnaire was designed to evaluate multiple domains simultaneously, systematically documenting a comprehensive array of life experiences and conditions from childhood to the present aiming to elucidate their cumulative effects on health. The MSE has been evaluated in multiple settings, demonstrating its predictive value for identifying preserved or accelerated aging across countries(1, 15, 26).

Education is evaluated through the number of years of formal education, the type of school attended (public or private), the geographical location of the school (rural or urban), and the overall quality of education received (from very bad to excellent), number of books at home during the first 10 years of life (from 0 to more than 200), and the educational achievement of the mother and father (from none to doctoral degree).
Food insecurity captures if the participant had to eat less (yes/no) or less healthy (yes/no) due to economic hardship across the lifespan (from 0 to 10, 35 to 45 years old, and recently), capturing the quality and consistency of food insecurity experienced by the participant across the lifespan.

Financial status assesses the participant's financial stability and ability to meet basic needs over different periods of their life (from 0 to 10, 35 to 45 years old, and recently). It measures the difficulty in covering basic needs, the sufficiency of income at the end of the month, the diversity of income sources, and the proportion of income spent on housing. Additionally, it gauges the participant's financial resilience in the event of lost income and health insurance (public, private, or none), offering a comprehensive view of their economic situation and security throughout their lifespan.

Assets evaluate the participant's access to essential household utilities and goods over different periods (from 0 to 10, 35 to 45 years old, and recently). It assesses whether the participant had access to electricity, radio, television, refrigerator, washing machine, landline telephone, water heater, indoor bathroom, running water, automobile, computer, internet, sound system, smartphone, and private room. Additionally, it measures the quantity of certain items like televisions, bathrooms, cars, computers, and private rooms the participant had during these

periods. This assessment provides a detailed view of the participant's material living conditions and how they have evolved throughout their lifespan.

Access to healthcare assesses the participant's difficulty in affording medical care across different life stages (from 0 to 10, 35 to 45 years old, and recently). It evaluates whether the participant experienced difficulty paying for medical services and explores specific barriers that may have prevented them from seeing a doctor (from not hard to very hard). These barriers include financial constraints, appointment availability, transportation issues, distance, lack of accompaniment, the COVID-19 pandemic, or socio-political events (yes/no). This assessment provides insights into the participant's access to healthcare and the factors influencing it throughout their lifespan.

Childhood labor investigates whether the participant worked before the age of 18 (yes/no) and the specific age range during which they started working (from 5 to 18 years old). It also delves into the reasons for working at a young age, including whether to meet personal needs, support family needs, save money, or learn a new skill (yes/no). This assessment provides insight into the participants' early exposure to work and the SES factors that may have influenced their decision to work during childhood or adolescence.

Subjective SES is evaluated by asking participants to reflect on their family's ability to meet basic needs and their financial situation from 0 to 10, 35 to 45 years old, and recently (from level 0 to 10).

Childhood experiences comprise the participant's early childhood environment, focusing on family structure, emotional support, and potential adverse experiences. It includes questions about the number of children in the household during the first 10 years, whether the participant had to leave school to support the household (yes/no), and the frequency of feeling loved by parents and receiving physical affection. Additionally, it explores conflicts within the family, such as fights between parents, parents-participant, and siblings. The questions also inquire about exposure to alcohol abuse, the organization of the household, and experiences of neglect during childhood (from never to frequently). This assessment provides a comprehensive view of the participant's early family life and its potential impact on their development.

Traumatic events evaluate the participant's exposure to traumatic events, violence, and maltreatment from 0 to 10, 11 to 24, 25 to 34, 35 to 45, 46 to 65 years old, less than a year, and recently. It includes questions about the loss of siblings or children before the age of 18, experiences of political violence and repression, and exposure to theft or robbery both inside and outside the home. It also examines the participants' involvement in or witness to accidents, situations where they feared for their lives, and encounters with dead or injured individuals. Furthermore, the questions explore experiences of physical violence, being attacked with a weapon, humiliation, restrictions on personal freedom, neglect, and financial control. Additionally, the set assesses instances where the participant was pressured to leave their property or coerced into sexual activity (yes/no). These experiences are considered in relation to different individuals (family members, friends, caregivers, strangers) (yes/no). This comprehensive assessment aims to capture the depth and breadth of the participant's exposure to adverse and potentially traumatic experiences throughout their life.

Relationships assess the participant's frequency of contact with loved ones and experiences of being treated with less respect across different stages of life and in recent times. It measures how often the participant interacts with close family or friends, ranging from less than once a week to more than five times a week. Additionally, it explores the frequency of disrespect or mistreatment experienced during various life stages (0 to 10, 11 to 24, 25 to 34, 35 to 45, 46 to 65 years old, less than a year, and recently). These experiences are categorized by how often they occur, ranging from almost daily to less than once a year or never. This assessment provides insights into the participant's social connections and the prevalence of negative interpersonal experiences throughout their life.

Scoring

The questionnaire employs various response formats, including Likert scales, multiple-choice questions (Yes/No), and free text fields. Privacy is prioritized, with the most sensitive questions, such as those about experiences of violence, abuse, and discrimination, answered individually by the participant (without the presence of the caregiver). We scored the questionnaire by creating composite variables by grouping items based on expert consensus on the similarity of the assessed domains. After excluding items each variable was min–max scaled between 0 and 1, with 0 and 1 indicating lower and higher levels of adversity of social exposome. Then, the dimension scores for each participant were obtained by averaging the value of the total number of variables per category (education, food insecurity, financial status, assets, access to healthcare, childhood labor, subjective SES, childhood experiences, traumatic events, and relations) to produce a score between 0 and 1. Confirmatory factor analyses showed a good model fit for the dimensions indicating that the dimension composite scores accurately represent the latent construct and consistently capture the intended dimensions.

Supplementary Information 5. Brain-age gap estimation

We estimated brain age using modality-specific models built from T1-weighted structural MRI (structural clock) and resting-state fMRI (functional clock). For each modality, the brain-age gap (BAG) was defined as predicted age minus chronological age (BAG < 0: delayed; BAG > 0: accelerated brain aging)(3, 4, 7). We obtained a third, combined BAG as the average of the structural and functional BAGs. Structural and functional models were trained using Light Gradient Boosting Machine (LightGBM)9 with nested 10-fold cross-validation; the combined score involved no additional training. To reduce site/scanner variability, we implemented intra-subject harmonization by min–max scaling the target (BAG) within scanner model(31, 50), ensuring consistent ranges across scanners.

Structural MRI (T1)

Structural preprocessing followed the CAT12 voxel-based morphometry pipeline (MATLAB; CAT12 v2.9; https://neuro-jena.github.io/cat/): bias-field correction, denoising, skull stripping, tissue segmentation, and normalization to Montreal Neurological Institute (MNI) space at 1.5 mm isotropic resolution. We then extracted gray-matter volume (GMV) based on 116 regions of interest (ROIs) using the Automated Anatomical Labeling (AAL116) (47) atlas and normalized regional GMV by total intracranial volume (TIV). All structural features were standardized (z-scored) prior to modeling to ensure comparability.

Resting-state fMRI

rs-fMRI preprocessing used the fMRIPrep toolbox (v22.0.2; https://fmriprep.org/) for motion correction, co-registration, spatial normalization, and confound estimation. We performed additional denoising with the CONN toolbox (v22), including 6×6×6 mm Gaussian smoothing, regression of WM/CSF/motion confounds, and scrubbing, and applied a 0.008–0.09 Hz band-pass filter. Regional time series were extracted from AAL116 (Python/Nilearn), producing one BOLD signal per ROI.

Functional features: higher-order interactions

From the parcellated rs-fMRI time series, we computed higher-order functional interactions using the organizational information metric (Ω; O-info)(49), a multivariate extension of Shannon's mutual information. For any ROI pair (i, j), Ω quantifies whether their relationship is redundancy-dominant ($\Omega > 0$) or synergy-dominant ($\Omega < 0$) with respect to the rest of the brain; entropy terms were estimated via a Gaussian-copula approach. The result is a symmetric 116×116 Ω-matrix (6,670 features). We retained the 3,321 connections corresponding to an 82×82 subset of AAL regions. For modeling, we vectorized the upper triangle (excluding the diagonal) into a feature vector ("Ω-features") representing higher-order connectivity.

Model optimization and nested cross-validation. Structural clock (MRI-T1)

Inputs were z-scored AAL116 GMV features (TIV-normalized). We trained a LightGBM regressor under nested 10-fold CV: an outer loop (10 folds) to provide unbiased test estimates, and an inner loop (9 folds) for hyperparameter tuning via grid search (learning rate 0.01–0.1; max depth 3–7; estimators 100–200). For each outer fold, we trained on the inner-selected configuration and predicted the held-out fold, yielding fold-wise predictions later concatenated to compute BAG (predicted minus chronological age).

Functional clock (rs-fMRI)

Inputs were Ω-features derived from the subject's 116×116 Ω-matrix. We used the same LightGBM and nested 10-fold CV scheme and hyperparameter grid as for the structural model, generating out-of-fold predictions subsequently converted to BAG.

Combined clock: Multimodal brain-age gap (BAG) estimation

After fitting two modality-specific BAG models—one from structural gray-matter volumes extracted from T1-MRI and another from rs-fMRI functional connectivity—we derived a combined BAG as the average of these estimates. By convention, BAG is defined as predicted minus chronological age, with negative values indicating delayed and positive values indicating accelerated brain aging. For participants with both modalities available, the combined BAG equals the arithmetic mean of the structural and functional BAGs. No additional model training or nested cross-validation was applied to the combined score, since it is computed directly from the modality-specific outputs (each obtained using LightGBM and nested 10-fold cross-validation). To reduce site/scanner variability, we applied an intra-subject harmonization step—min–max scaling of BAG within scanner model—and we did not apply age-bias correction in the main analyses, as post hoc age-bias adjustments can inflate apparent performance (e.g., r and R^2) and obscure true variability.

As noted above, to mitigate site/scanner variability we applied min–max scaling of the BAG within scanner model, constraining values to a fixed range per scanner and ensuring consistent minima and maxima across devices. This intra-subject harmonization reduces scanner-driven dispersion in BAG while preserving individual differences relevant for biological interpretation. All primary results reflect raw out-of-fold predictions from nested CV.

Supplementary Information 6. Model evaluation

Model performance was assessed in each outer fold and metrics averaged across the 10 folds. Metrics included the coefficient of determination (R^2; proportion of variance in chronological age explained by the model); the Mean Directional Error (MDE; the average sign of the prediction error, sign [predicted age − chronological age]); Mean Absolute Error (MAE; the average absolute difference between predicted and chronological age); Cohen's f^2 (effect size, with > 0.35 indicating a large effect); and P values (obtained via 5,000-iteration non-parametric permutation tests). To interpret models, we computed Shapley Additive Explanations (SHAP) values to rank ROI-level feature importance.
